# The Great Oxygenation Event as a consequence of ecological dynamics modulated by planetary change

**DOI:** 10.1038/s41467-021-23286-7

**Published:** 2021-06-28

**Authors:** Jason Olejarz, Yoh Iwasa, Andrew H. Knoll, Martin A. Nowak

**Affiliations:** 1 Department of Organismic and Evolutionary Biology, Harvard University, Cambridge, MA USA; 2 Department of Bioscience, School of Science and Technology, Kwansei Gakuin University, Sanda-shi, Hyogo Japan; 3 Department of Mathematics, Harvard University, Cambridge, MA USA

**Keywords:** Microbial ecology, Palaeoecology, Biogeochemistry, Atmospheric chemistry

## Abstract

The Great Oxygenation Event (GOE), ca. 2.4 billion years ago, transformed life and environments on Earth. Its causes, however, are debated. We mathematically analyze the GOE in terms of ecological dynamics coupled with a changing Earth. Anoxygenic photosynthetic bacteria initially dominate over cyanobacteria, but their success depends on the availability of suitable electron donors that are vulnerable to oxidation. The GOE is triggered when the difference between the influxes of relevant reductants and phosphate falls below a critical value that is an increasing function of the reproductive rate of cyanobacteria. The transition can be either gradual and reversible or sudden and irreversible, depending on sources and sinks of oxygen. Increasing sources and decreasing sinks of oxygen can also trigger the GOE, but this possibility depends strongly on migration of cyanobacteria from privileged sites. Our model links ecological dynamics to planetary change, with geophysical evolution determining the relevant time scales.

## Abstract (web summary)

The Great Oxygenation Event (GOE) 2.4 billion years ago is believed to have been critical for the evolution of complex life. Here, Olejarz et al. propose a model suggesting that competition between major bacterial groups could have triggered the GOE in a feedback loop with geophysical processes.

## Introduction

**Fig. 1:**
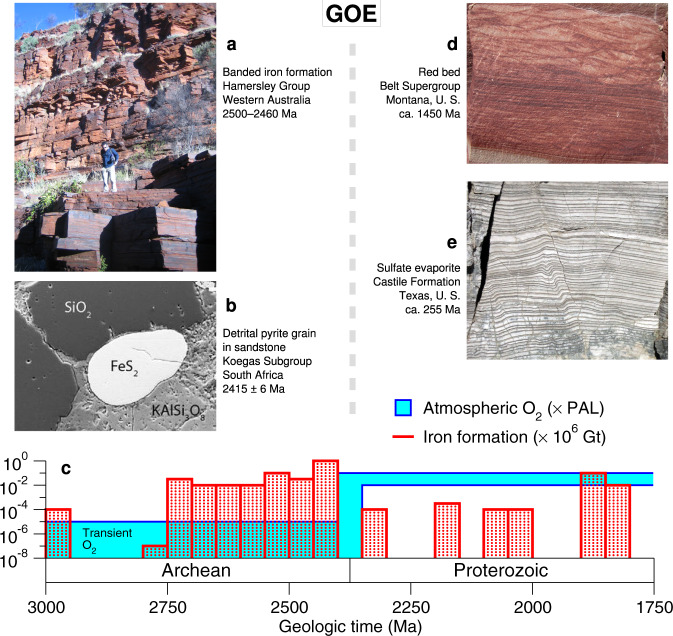
Evidence for the GOE. Multiple lines of geologic and geochemical evidence support the view that oxygen gas first became a permanent component of Earth’s atmosphere and surface ocean ca. 2.4 billion years ago. Sedimentary iron formation (**a**), which requires transport of ferrous iron through the ocean, is abundant in successions that predate the GOE but uncommon afterward (**c**, with resurgences around 1900–1850 and 715–660 Ma). Similarly, redox-sensitive minerals such as pyrite (FeS_2_) occur in detrital facies before the GOE (**b**) but not afterward. In contrast, red beds (**d**) and sulfate salts (**e**), which bespeak O_2_ in surface environments, have the opposite time distribution, gaining prominence only after the GOE. It is estimated that atmospheric pO_2_ increased from <10^−5^ to 1–10% of PAL at this time (**c**). (Data on iron formations in (**c**) are taken from Bekker et al.^4^. The blue shaded region denoting atmospheric O_2_ levels is only notional, as it is possible that atmospheric pO_2_ dropped below 1% of PAL during the Proterozoic^1,2^.).

The Great Oxygenation Event (GOE), ~2.4 billion years ago, records a major turning point in the history of our planet. While pO_2_ may have fluctuated during the GOE^1,2^, its results are clear: multiple lines of geological and geochemical evidence document the initial rise of O_2_ to permanent prominence in the atmosphere and surface ocean^3^. These include (1) a sharp drop in iron formation deposition^4^, (2) the appearance of red beds in continental sedimentary successions^5^ and calcium sulfates in marine environments^6^, (3) the retention of iron in ancient weathering horizons^7^, (4) the loss of detrital uraninite and other redox-sensitive minerals from fluvial and deltaic sandstones^8^, and (5) the loss of a mass-independent sulfur-isotopic signature best explained in terms of photochemical reactions in an essentially oxygen-free atmosphere^9,10^. Detrital pyrite and uraninite suggest that, prior to the GOE, pO_2_ was <1.6 × 10^−4^ of present atmospheric levels (PAL)^11^, whereas modeling of mass-independent S-isotopic fractionation limits Archean pO_2_ to ~10^−5^ of PAL or lower^9,12,13^. Figure 1 summarizes the main geological and geochemical lines of evidence for the GOE.

While there is consensus on when the biosphere began to accumulate oxygen, debate continues about the physical and/or biological drivers of this transition. As emphasized by Kasting^14^, the GOE required an increase in the rate of oxygen production and/or a decrease in rates of oxygen consumption. Cyanobacterial photosynthesis is generally accepted as the key source of oxygen, but many models to explain the GOE tacitly assume that cyanobacteria were abundant prior to the GOE and so rely on physical events as the proximal drivers of environmental state change^14–16^. Proposed physical drivers include hydrogen escape to space associated with the photodissociation of H_2_O and CH_4_ in the upper atmosphere^17,18^, a temporal shift to more oxidized volcanic gases as subaerial volcanism increased along with craton expansion^19,20^, or a decrease in hydrothermal iron fluxes into the ocean as vent temperatures declined^19,21,22^. Limited phosphorus availability in Archean oceans has also been suggested as limiting rates of photosynthesis and carbon burial^23–25^.

Goldblatt et at.^26^ considered that oxygenic photosynthesis was well established prior to the GOE but that atmospheric methane oxidation suppressed oxygen levels. They modeled a low-level steady state of oxygen and a high-level steady state—the latter being characterized by an ozone layer that shields the troposphere from ultraviolet radiation, limiting the rate of methane oxidation. These steady states of oxygen can overlap, resulting in bistability and hysteresis. Also related to methane oxidation, Konhauser et al.^27^ proposed a coupled biological-geological driver for the GOE, concluding that a reduced flux of nickel to the oceans in the late Archean limited methanogen activity, thereby capping the supply of biogenic methane.

Other investigations of the carbon isotope record have argued that the *δ*^13^C value of marine carbonate increased from the Archean into the Proterozoic^28,29^, raising the possibility that biological innovations acted as triggers for the GOE. In contrast to geophysical models, Ward et al.^30^ modeled the GOE as a direct response to the origin of oxygenic photosynthesis. This implicitly assumes that cyanobacteria hold an inherent selective advantage over anoxygenic photoautotrophs, but the dominance of anoxygenic photosynthetic bacteria in modern environments where light is present but oxygen is not (e.g., ref. ^31^) casts substantial doubt on this assumption. The competitive advantage of anoxygenic photosynthetic bacteria over cyanobacteria may relate to the lower metabolic cost of deriving electrons from donors other than water, or it may reflect the direct inhibition of oxygenic photosynthesis by sulfide^32^ or ferrous iron^33^. In the presence of sulfide, some cyanobacteria can shut down Photosystem II and use H_2_S as an electron donor for anoxygenic photosynthesis^34,35^. The inhibitory effect of Fe^2+^ is less clear, as Ward et al.^36^ show that cyanobacteria live in hot spring waters that contain both ferrous iron and modest amounts of oxygen. Where Fe^2+^ was present and O_2_ was absent, however, cyanobacteria were not ecologically important^36^. Moreover, a growing array of geochemical data is interpreted as evidence for “whiffs of oxygen”—spatially and temporally limited oxygen oases in the Archean biosphere—requiring oxygenic photosynthesis hundreds of millions of years before the GOE^37–39^.

A natural scenario, then, is that cyanobacteria were present but relatively scarce well before the GOE, rising to global ecological prominence coincident with the environmental transformation. Jones et al.^40^ argued that as the ratio of alternative electron donors (they focused on Fe^2+^, but it is the sum of non-H_2_O electron donors that is key) to phosphorus declined through time, environmental opportunities for oxygenic photoautotrophs increased. Knoll and Nowak^41^ developed a simple mathematical model to describe the competition between anoxygenic photosynthetic bacteria and cyanobacteria on a changing Earth. Their model keeps track of the abundances of anoxygenic photosynthetic bacteria, cyanobacteria, ferrous iron, and oxygen. Simulations revealed that the GOE can be triggered as the planetary removal rate of oxygen from the atmosphere declines, although that primitive model was not analytically solved. Ozaki et al.^42^ used both modeling and experimental evidence, arguing once again that oxygenic photoautotrophs evolved long before the GOE, but that geochemical conditions throughout the Archean favored primary production by anoxygenic photosynthetic bacteria. They further argued that anoxygenic photoferrotrophs, being well adapted to low-light conditions, inhabited the bottom of the photic zone, thereby diminishing the supply of upwelling nutrients to cyanobacteria (see also ref. ^43^). This control on the proliferation of oxygenic bacteria would have remained in place as long as there was a sufficiently large supply of reducing agents from the deep ocean.

Here, we advance the model of Knoll and Nowak^41^ by additionally keeping track of the abundance of phosphate, because the amount of ferrous iron or other alternative electron donors relative to phosphate influences the competition between anoxygenic photosynthetic bacteria and cyanobacteria. We also properly account for the loss of ferrous iron due to the proliferation of anoxygenic photoautotrophs, and we correctly incorporate loss of dioxygen due to iron oxidation. We can now investigate the effects of a declining influx of iron and an increasing influx of phosphate as consequences of planetary change, while concurrently probing the influences of biological innovations and of changes in sources and sinks of oxygen. Our model thus focuses consideration of the GOE on interactions between the physical and biological Earth.

## Results

Based on the present-day distribution of photosynthetic bacteria^31^, we assume a competitive advantage for anoxygenic photosynthetic bacteria in early environments where electron donors such as Fe^2+^, H_2_S, or H_2_ were present. We also assume the contemporaneous existence of environments where cyanobacterial populations could thrive, providing a seedbed for migration. Non-marine waters provide an example of the latter, supported by the branching of non-marine taxa from basal nodes in cyanobacterial phylogenies^44,45^ and also by the presence of stromatolites in Archean lacustrine successions^46^, despite the likelihood that many Archean lakes and rivers had low levels of potential electron donors such as Fe^2+^ and H_2_S^47^.

Following Jones et al.^40^ and Ozaki et al.^42^, we use Fe (iron) and P (phosphorus) to represent the environment, which is similar to the H_2_ and P employed in other studies^48,49^. The logic of this choice is that in Archean oceans, Fe^2+^ is thought to have been the principal electron donor for anoxygenic photosynthesis^50,51^, whereas P governed total rates of photosynthesis. (Kasting^14^ argued that H_2_ was key to photosynthesis on the early Earth, a view supported by low iron concentrations in some early Archean stromatolites^52^.). In any event, under the conditions of low P availability thought to have characterized early oceans^25,40,49,53–55^, anoxygenic photosynthesis would have depleted limiting nutrients before alternative electron donors were exhausted. In consequence, rates of photosynthetic oxygen production would be low. As iron availability declined and/or P availability increased, the biosphere would inevitably reach a point where P would remain after Fe^2+^ had been depleted, expanding the range of environments where cyanobacteria are favored by natural selection^42^.

Our model keeps track of the abundances of anoxygenic photosynthetic bacteria (APB), *x*_1_, cyanobacteria, *x*_2_, and three crucial chemicals: iron(II) (Fe^2+^), *y*_1_, phosphate (PO_4_^3−^), *y*_2_, and dioxygen (O_2_), *z*. Both types of bacteria require phosphate for reproduction. APB needs iron(II) (or some other suitable reductant) as an electron donor in photosynthesis. The following five equations describe the reproduction and death of APB and of cyanobacteria as well as the dynamics of iron(II), phosphate, and dioxygen:

1 $${\rm{APB}}:\ {\dot{x}}_{1} 	={x}_{1}{y}_{1}{y}_{2}-{x}_{1}+{u}_{1}\\ {\rm{Cyano}}:\ {\dot{x}}_{2} 	=c{x}_{2}{y}_{2}-{x}_{2}+{u}_{2}\\ {{\rm{Fe}}}^{2+}:\ {\dot{y}}_{1} 	={f}_{1}-{y}_{1}-{x}_{1}{y}_{1}{y}_{2}-{y}_{1}z\\ {{\rm{PO}}_{4}}^{3-}:\ {\dot{y}}_{2} 	={f}_{2}-{y}_{2}-{x}_{1}{y}_{1}{y}_{2}-{x}_{2}{y}_{2}\\ {{\rm{O}}}_{2}:\ \dot{z} 	=a{x}_{2}{y}_{2}-bz-{y}_{1}z$$

Here, we have omitted to write symbols for those rate constants that, for understanding the GOE, can be set to one without loss of generality (Supplementary Note 1). Each remaining rate constant is a free parameter. Equations (1) thus satisfy redox balance by construction. We are left with a system that has five main parameters: *c* specifies the rate of reproduction of cyanobacteria; *f*_1_ and *f*_2_ denote the rates of supply of iron(II) and phosphate, respectively; *a* denotes biogenic production of oxygen; *b* denotes geochemical consumption of oxygen. Note that iron(II) and phosphate are also removed by geochemical processes at a rate proportional to their abundance. In addition, iron(II) is used up during anoxygenic photosynthesis, and iron(II) reacts with oxygen and is thereby removed from the system. Phosphate is used up during the growth of APB and cyanobacteria. (We investigate extensions of the model that incorporate bounded bacterial growth rates and organic carbon in Supplementary Note 2 and Supplementary Note 3, respectively.)

We posit iron(II) as the primary electron donor for anoxygenic photosynthesis, and for simplicity of presentation, we refer to *y*_1_ and *f*_1_ in this context. However, as noted above, *y*_1_ and *f*_1_ can similarly represent the abundances and influxes of other alternative electron donors, especially dihydrogen (H_2_)^56,57^ and hydrogen sulfide (H_2_S)^58^. Our model, its analytical solution, and the conclusions that follow hold equally well by considering any of these electron donors or all together.

We also include small migration rates, *u*_1_ and *u*_2_, which allow for the possibility that APB and cyanobacteria persist in privileged sites from which they can migrate into the main arena of competition. On the Archean Earth, these parameters could have been affected by the flow of water and by surface winds. For the mathematical analysis presented in the main text, we assume that these rates are negligibly small.

The GOE represents the transition from a world dominated by APB (Equilibrium *E*_1_) to one that is dominated by cyanobacteria (Equilibrium *E*_2_) (Figs. S1, S2). On a slowly changing planet, the abundances of APB and cyanobacteria and of the three chemicals are approximately in steady state. Therefore, we consider the fixed points of Eqs. (1).

### Pure equilibria

In the absence of APB and cyanobacteria, the abiotic equilibrium abundances of iron(II) and of phosphate are given by *f*_1_ and *f*_2_, respectively, and there is no oxygen in the system. If *f*_1_*f*_2_ > 1, then APB can emerge. Subsequently, the system settles to Equilibrium *E*_1_, where only APB are present and there is still no oxygen. *E*_1_ is stable against invasion of cyanobacteria if

2 $${f}_{1}-{f}_{2}\,> \,\frac{(c+1)(c-1)}{c}.$$

This condition can be fulfilled if the influx of iron, *f*_1_, is large enough, or if the influx of phosphate, *f*_2_, is small enough. The term on the right-hand side of the inequality is an increasing function of the reproductive rate, *c*, of cyanobacteria.

If *cf*_2_ > 1, then the system admits another equilibrium, *E*_2_, where only cyanobacteria are present and oxygen is abundant. Equilibrium *E*_2_ is stable against invasion of APB if

3 $$a(c{f}_{2}-1)\,> \,(b+c)({f}_{1}-c).$$

The left-hand side of the inequality is positive. If the right-hand side is negative (that is, if *f*_1_ < *c*), then the condition certainly holds. If the right-hand side is positive, then the condition can be fulfilled if the influx of phosphate, *f*_2_, is large enough, or if the production of oxygen, *a*, is large enough. In other words, the dominance of cyanobacteria after the GOE can be guaranteed by a sufficiently large supply of phosphate or sufficiently large production of oxygen. It may or may not be possible for the proportional removal rate of oxygen, *b*, to become small enough for the condition to be fulfilled.

### Mixed equilibrium

If Conditions (2) and (3) are either both satisfied or both not satisfied, then the system also admits an interior equilibrium, $\hat{E}$. If Conditions (2) and (3) are both satisfied, then Equilibrium $\hat{E}$ is unstable; if those conditions are both not satisfied, then Equilibrium $\hat{E}$ is a stable mixed equilibrium where both types of bacteria coexist. Equilibrium $\hat{E}$ is characterized by the stable coexistence of APB and cyanobacteria if

4 $$b\,> \,c(a-1).$$

Condition (4) is understood as follows. If *b* is sufficiently large, then there is not enough atmospheric oxygen for rusting to render *E*_2_ stable against invasion of APB before *E*_1_ loses stability; the result is stable coexistence. But if *b* is sufficiently small, then rusting causes *E*_2_ to become stable before *E*_1_ becomes unstable. The critical value of *b* therefore depends on the input of atmospheric oxygen for Equilibrium *E*_2_; it is an increasing function of the reproductive rate of cyanobacteria and of their rate of production of oxygen.

If *a* < 1, then bistability is not possible. In this case, for Equilibrium *E*_2_, dioxygen is depleted by rusting before there is any significant loss of iron(II). As a result, *E*_2_ cannot gain stability before *E*_1_ loses stability, regardless of the values of *b* or *c*.

**Fig. 2:**
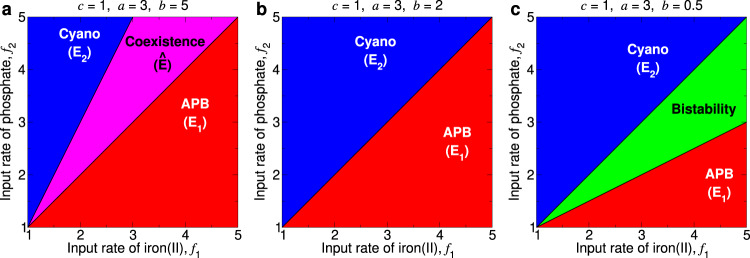
The stability properties of Equilibrium *E*_1_ (APB dominate) and Equilibrium *E*_2_ (cyanobacteria dominate) depend on the input rate of iron(II), *f*_1_, and the input rate of phosphate, *f*_2_. High values of *f*_1_ and low values of *f*_2_ promote stability of *E*_1_ and instability of *E*_2_. Low values of *f*_1_ and high values of *f*_2_ promote instability of *E*_1_ and stability of *E*_2_. **a** If the proportional consumption rate of oxygen, *b*, is large, then intermediate values of *f*_1_ and *f*_2_ lead to both *E*_1_ and *E*_2_ being unstable, with Equilibrium $\hat{E}$ corresponding to stable coexistence. **b** For an intermediate value of *b*, either *E*_1_ is stable with *E*_2_ unstable, or *E*_1_ is unstable with *E*_2_ stable. **c** If *b* is small, then intermediate values of *f*_1_ and *f*_2_ lead to both *E*_1_ and *E*_2_ being stable.

Figure 2 shows, for different values of *b*, the behavior of the system as a function of *f*_1_ and *f*_2_.

### Transition from Equilibrium *E*_1_ to Equilibrium *E*_2_

The transition between Equilibria *E*_1_ and *E*_2_ can be achieved by reducing the supply of iron(II), *f*_1_, since such a reductant is required for anoxygenic photosynthesis. When this happens, we lose the stability of *E*_1_ and gain the stability of *E*_2_.

**Fig. 3:**
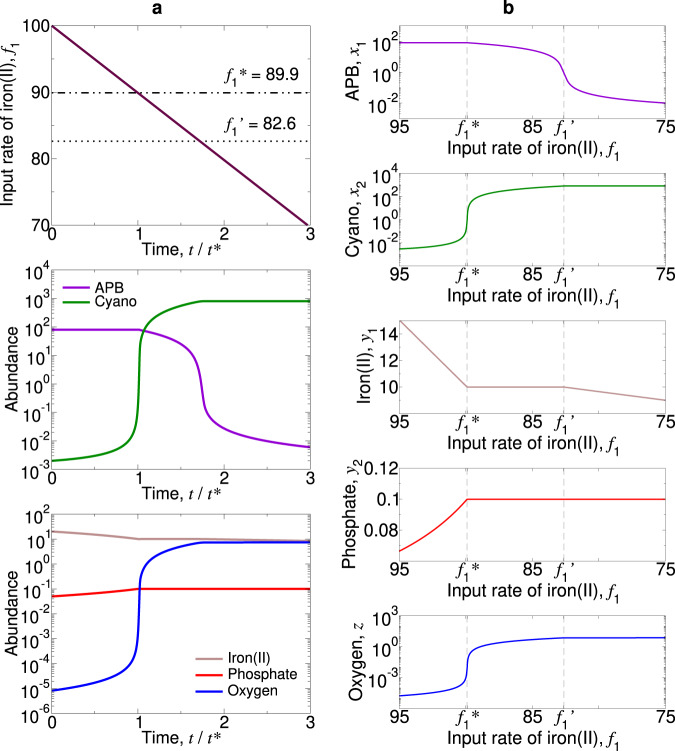
The GOE can be triggered by a decline in the influx of iron(II) and is gradual if *b* > *c*(*a* − 1). Equilibrium *E*_1_ (APB dominate) loses stability and Equilibrium *E*_2_ (cyanobacteria dominate) gains stability when *f*_1_ drops below ${f}_{1}^{* }$ and $f_1^{\prime}$, respectively. We set *f*_2_ = 80, *c* = 10, *a* = 10, *b* = 100, and *u*_1_ = *u*_2_ = 10^−3^. **a** We simulate Eqs. (8) from Supplementary Note 1 with *α*_1_ = *α*_2_ = *β*_1_ = *β*_2_ = 1, and we set *f*_1_ = 100 − 40(*t*/10^5^). t^*^ denotes the time at which Equilibrium *E*_1_ loses stability. **b** There is stable coexistence of both types of bacteria for $f_1^{\prime} \,<\,{f}_{1}\,<\,{f}_{1}^{* }$.

**Fig. 4:**
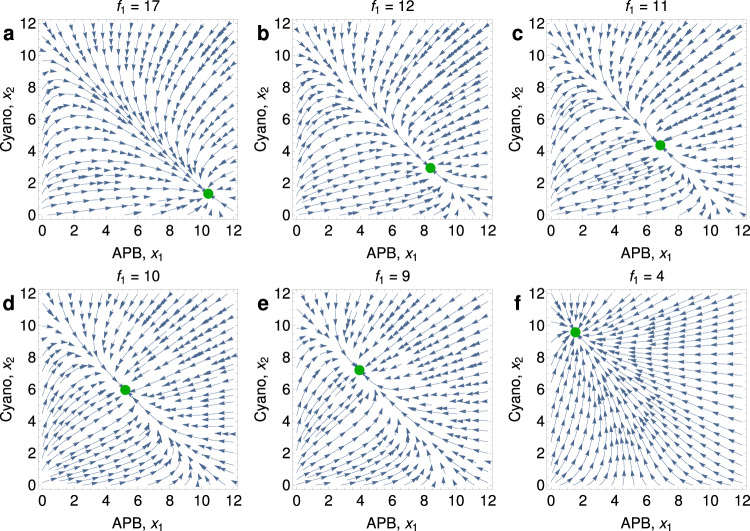
Phase portraits of Eqs. (14) from Supplementary Note 1 illustrating a gradual transition are shown for six distinct values of *f*_1_, the influx rate of iron(II). For values of *f*_1_ = 17 (**a**), 12 (**b**), 11 (**c**), 10 (**d**), 9 (**e**), and 4 (**f**), the stable equilibrium (green dot) moves continuously from a world that is dominated by APB to one that is dominated by cyanobacteria. Parameter values are *f*_2_ = 10, *c* = 1, *a* = 10, *b* = 12, *u*_1_ = *u*_2_ = 1, and *α*_1_ = *α*_2_ = 1. The GOE is gradual.

The transition is gradual if *b* > *c*(*a* − 1). Figure 3 shows gradual oxygenation due to decreasing *f*_1_. In this case, the transition occurs via the mixed equilibrium, $\hat{E}$, where both types of bacteria coexist (Fig. 4). A subsequent increase in *f*_1_ can cause APB to regain dominance (Fig. S3a).

**Fig. 5:**
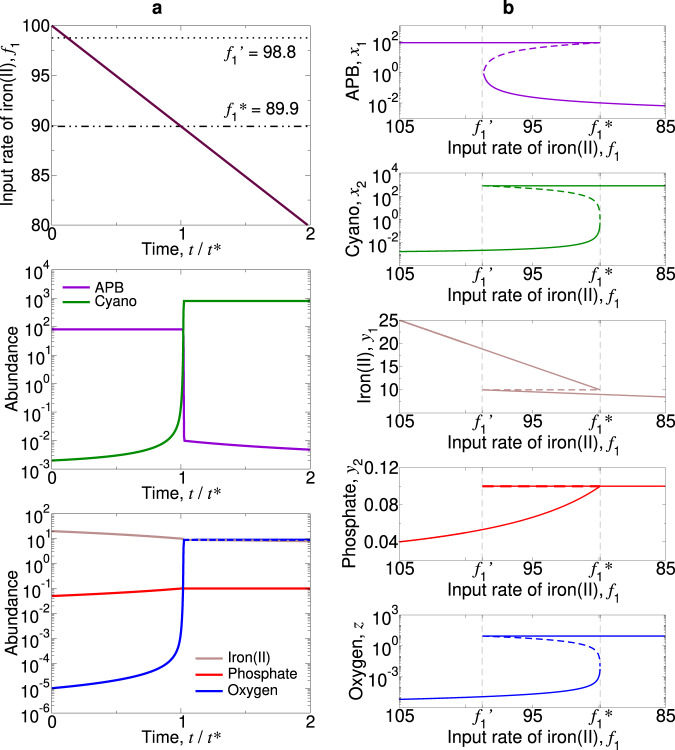
The GOE can be triggered by a decline in the influx of iron(II) and is sudden if *b* < *c*(*a* − 1). Equilibrium *E*_2_ (cyanobacteria dominate) gains stability and Equilibrium *E*_1_ (APB dominate) loses stability when *f*_1_ drops below $f_1^{\prime}$ and ${f}_{1}^{*}$, respectively. We set *f*_2_ = 80, *c* = 10, *a* = 10, *b* = 80, and *u*_1_ = *u*_2_ = 10^−3^. **a** We simulate Eqs. (8) from Supplementary Note 1 with *α*_1_ = *α*_2_ = *β*_1_ = *β*_2_ = 1, and we set *f*_1_ = 100 − 40(*t*/10^5^). t^*^ denotes the time at which Equilibrium *E*_1_ loses stability. **b** Bifurcation plots reveal bistability for ${f}_{1}^{* }\,<\,{f}_{1}\,<\,{f}_{1}^{\prime}$.

**Fig. 6:**
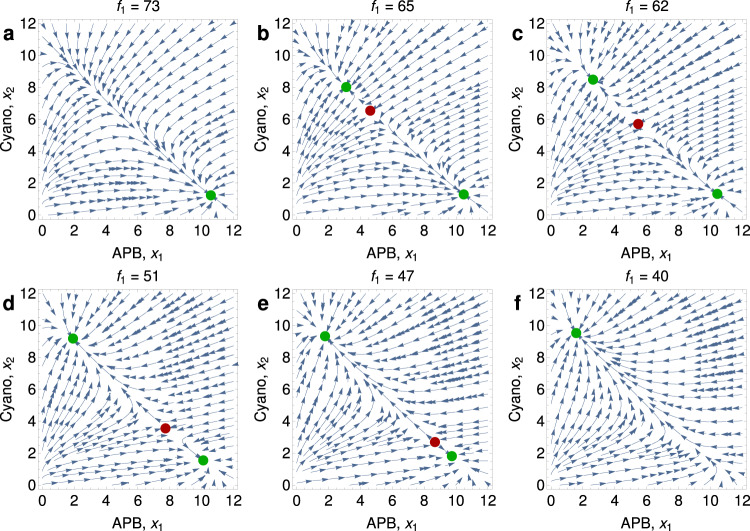
Phase portraits of Eqs. (14) from Supplementary Note 1 illustrating a sudden transition are shown for six distinct values of *f*_1_, the influx rate of iron(II). For *f*_1_ = 73 (**a**), there is a single stable equilibrium (green dot) describing a world dominated by APB. For values of *f*_1_ = 65 (**b**), 62 (**c**), 51 (**d**), and 47 (**e**), there is a second stable equilibrium (green dot) describing the dominance of cyanobacteria, and in addition, there is an unstable equilibrium (red dot). The unstable equilibrium moves as the value of *f*_1_ changes. For *f*_1_ = 40 (**f**), the only stable equilibrium is the one where cyanobacteria dominate. Parameter values are *f*_2_ = 10, *c* = 1, *a* = 10, *b* = 1, *u*_1_ = *u*_2_ = 1, and *α*_1_ = *α*_2_ = 1. The GOE is triggered by a saddle-node bifurcation and is sudden.

Alternatively, if *b* < *c*(*a* − 1), then the transition is sudden (i.e., discontinuous). Figure 5 shows rapid oxygenation due to decreasing *f*_1_. In this case, *E*_2_ is already stable before *E*_1_ loses stability (Fig. 6). This results in bistability and hysteresis: Once the world is dominated by cyanobacteria, moderate fluctuations in the supply rate of iron would no longer change the status quo (Fig. S3b).

The effects of increasing *f*_2_ are nearly identical to those of decreasing *f*_1_. Increasing the supply of phosphate results in loss of stability of *E*_1_ and gain of stability of *E*_2_. This is because as *f*_2_ rises, APB proliferate, inducing a concomitant depletion of iron reserves. The transition is gradual if *b* > *c*(*a* − 1) (Fig. S4) or sudden if *b* < *c*(*a* − 1) (Fig. S5). The critical values of *f*_1_ and *f*_2_ are robust to changes in *u*_2_ (Figs. S6a, S6b).

Another possibility is that the GOE resulted from an increase in the parameter *c*, which denotes the reproductive rate of cyanobacteria, as affected by biological mutations. We cannot exclude the possibility that cyanobacterial performance and, therefore, primary production increased as a function of genetic innovations; however, the observation that even today oxygenic photosynthesis by cyanobacteria is limited when alternative electron donors are present places limits on such speculation. The parameter *c* could also be affected by geophysical or geochemical properties unrelated to oxygen consumption and independent of iron(II) or phosphate flux, such as temperature, pH, salinity, or availability of trace nutrients or other resources. The transition can be gradual (Fig. S7) or sudden (Fig. S8), depending on whether *b* > *c*(*a* − 1) or *b* < *c*(*a* − 1) when *c* is such that Equilibrium *E*_1_ becomes unstable. Similar to the critical values of *f*_1_ and *f*_2_, the critical value of *c* for triggering a GOE is robust to changes in *u*_2_ (Fig. S6c).

Yet another possibility is that the GOE was triggered by an increase in parameter *a*, which measures the production rate of oxygen (Fig. S9), or by a reduction in parameter *b*, which denotes the proportional consumption rate of oxygen (Fig. S10). For this transition to occur, however, it is essential that *u*_2_ is sufficiently large. Moreover, the critical values of *a* and *b* are strongly dependent on the magnitude of *u*_2_. If *a* is not large enough, then it is not possible for a reduction in *b* to trigger a GOE, regardless of how small *b* becomes (Fig. S11).

A GOE resulting from an increase in *a* or a decrease in *b* is necessarily sudden. This is because as *a* rises or *b* declines, Equilibrium *E*_2_, which is characterized by abundance of oxygen, may eventually gain stability, while Equilibrium *E*_1_ remains stable. If *a* becomes sufficiently large or *b* becomes sufficiently small, then *E*_1_ may cease to exist, and a saddle-node bifurcation results in rapid oxygenation.

### Effects of migration rate

The migration rates, *u*_1_ and *u*_2_, have negligible effects on the abundance of APB for Equilibrium *E*_1_ and on the abundances of cyanobacteria and oxygen for Equilibrium *E*_2_. The principal effects of the migration rates are to determine the abundance of APB for Equilibrium *E*_2_ and the abundances of cyanobacteria and oxygen for Equilibrium *E*_1_. As such, *u*_1_ and *u*_2_ control the magnitude of the decline in APB across the GOE and the magnitude of the rise in cyanobacteria and oxygen across the GOE (Figs. S12, S13).

## Discussion

Our analytical investigation of ecological dynamics indicates that a switch in ecological dominance from APB to cyanobacteria would have been sufficient to spawn the GOE. Accordingly, the competitive advantage of cyanobacteria, *c*, the influx of suitable reductants, *f*_1_, and the influx of phosphate, *f*_2_, that appear as parameters in Condition (2) are key considerations for determining when the GOE began. As extant cyanobacteria display low fitness in sunlit but anoxic environments, this observation must condition any proposed mechanisms by which *c* could have substantially increased around the time of the GOE. In contrast, a decrease in *f*_1_ and/or an increase in *f*_2_ comprise robust mechanisms for initiating the GOE. Decreasing Fe and increasing P fluxes to the oceans are both predicted by secular cooling of the mantle (and hydrothermal systems), continental emergence, and increasing oxidant supply as the GOE began^22,24,53–55^. Our analysis reveals the time at which the GOE began to be determined by the difference, *f*_1_ − *f*_2_, in these influxes. It does not depend on these influxes individually. This functional dependence is preserved under the assumption that bacterial growth rates are limited (Supplementary Note 2, Fig. S14), as are the possibilities of both gradual and sudden transitions (Figs. S15, S16). These results also hold when explicitly accounting for organic carbon (Supplementary Note 3, Fig. S17).

While prior investigations have focused heavily on sources and sinks of oxygen as potential drivers of the GOE, our analysis emphasizes that this possibility hinges critically on competition from cyanobacteria. If *u*_2_ was small, then sources and sinks of oxygen, *a* and *b*, were mostly irrelevant for initiating the GOE. There is a simple intuition behind this observation. If cyanobacteria were ecologically subordinate to APB and scarce in the Archean, then atmospheric levels of O_2_ would have remained low. *a* might have increased and *b* might have decreased—and fractional changes in these parameters could have been substantial—but when multiplied by low abundance of cyanobacteria, the absolute change in atmospheric O_2_ levels would also be small.

Our model of ecological dynamics is robust to a broad range of influences on primary production, oxygen generation, and oxygen consumption. Our study also emphasizes that it was not strictly geophysical processes or biological innovations that ushered in the GOE, but rather the interplay between Earth and life as populations adapted to a changing planet.

## Methods

We elaborate our mathematical model of ecological dynamics, its analytical solutions, the stability properties of its fixed points, and important considerations behind the GOE in Supplementary Note 1. Here, we provide an abbreviated account of our analysis and key findings.

### Ecological dynamics and fixed points

Equations (1) specify the ecological dynamics of APB and cyanobacteria. They set the foundation for our understanding of the GOE. To make progress analytically, we make two simplifying assumptions. First, we assume that, at any given time, Equations (1) are approximately in steady state. This is because *f*_1_, *f*_2_, *c*, *a*, and *b* change very slowly relative to the typical reproductive lifetimes of the bacteria. Second, we assume that *u*_1_ and *u*_2_ are both small. Equations (1) become

5 $$0 	=({\bar{y}}_{1}{\bar{y}}_{2}-1){\bar{x}}_{1}\\ 0 	=(c{\bar{y}}_{2}-1){\bar{x}}_{2}\\ 0 	={f}_{1}-{\bar{y}}_{1}-{\bar{x}}_{1}{\bar{y}}_{1}{\bar{y}}_{2}-{\bar{y}}_{1}\bar{z}\\ 0 	={f}_{2}-{\bar{y}}_{2}-{\bar{x}}_{1}{\bar{y}}_{1}{\bar{y}}_{2}-{\bar{x}}_{2}{\bar{y}}_{2}\\ 0 	=a{\bar{x}}_{2}{\bar{y}}_{2}-b\bar{z}-{\bar{y}}_{1}\bar{z}$$

Equations (5) admit a solution for which the equilibrium abundance of cyanobacteria is zero:

6 $${x}_{1}^{(1)} 	=\frac{{f}_{1}+{f}_{2}-\sqrt{{({f}_{1}-{f}_{2})}^{2}+4}}{2}\\ {x}_{2}^{(1)} 	=0\\ {y}_{1}^{(1)} 	=\frac{{f}_{1}-{f}_{2}+\sqrt{{({f}_{1}-{f}_{2})}^{2}+4}}{2}\\ {y}_{2}^{(1)} 	=\frac{{f}_{2}-{f}_{1}+\sqrt{{({f}_{1}-{f}_{2})}^{2}+4}}{2}\\ {z}^{(1)} 	=0$$

Equations (5) admit another solution for which the equilibrium abundance of APB is zero:

7 $${x}_{1}^{(2)} 	=0\\ {x}_{2}^{(2)} 	=c{f}_{2}-1\\ {y}_{1}^{(2)} 	=\frac{c({f}_{1}-b)-a(c{f}_{2}-1)+\sqrt{{[c({f}_{1}-b)-a(c{f}_{2}-1)]}^{2}+4b{c}^{2}{f}_{1}}}{2c}\\ {y}_{2}^{(2)} 	=\frac{1}{c}\\ {z}^{(2)} 	=\frac{a(c{f}_{2}-1)-c({f}_{1}+b)+\sqrt{{[c({f}_{1}-b)-a(c{f}_{2}-1)]}^{2}+4b{c}^{2}{f}_{1}}}{2bc}$$

Equations (5) also admit a solution for which the equilibrium abundances of cyanobacteria and APB are both nonzero:

8 $${\hat{x}}_{1} 	=\frac{q}{r}\\ {\hat{x}}_{2} 	=(b+c)\left(\frac{p}{r}\right)\\ {\hat{y}}_{1} 	=c\\ {\hat{y}}_{2} 	=\frac{1}{c}\\ \hat{z} 	=\left(\frac{a}{c}\right)\left(\frac{p}{r}\right)$$

Here, we have set

9 $$p=c[c-({f}_{1}-{f}_{2})]-1$$

10 $$q=(b+c)({f}_{1}-c)-a(c{f}_{2}-1)$$

11 $$r=b-c(a-1)$$

### Dynamical stability

Equilibrium *E*_1_, given by Eqs. (6), is stable if *p* < 0 and unstable if *p* > 0. From Eq. (9), setting *p* < 0 and rearranging, we obtain Condition (2). Equilibrium *E*_2_, given by Eqs. (7), is stable if *q* < 0 and unstable if *q* > 0. From Eq. (10), setting *q* < 0 and rearranging, we obtain Condition (3). Equilibrium $\hat{E}$, given by Eqs. (8), is stable if *r* > 0 and unstable if *r* < 0. From Eq. (11), setting *r* > 0 and rearranging, we obtain Condition (4).

### Timing and nature of the GOE

The GOE corresponds to a transition between Equilibrium *E*_1_ and Equilibrium *E*_2_.

One possibility is that the GOE is gradual. Initially, *E*_1_ is stable, while *E*_2_ is unstable. When *E*_1_ loses dynamical stability, a stable interior fixed point, given by Eqs. (8), (9), (10), and (11), appears near *E*_1_ in phase space. As parameter values become more favorable to cyanobacteria, the interior fixed point moves toward *E*_2_ in phase space, and oxygenation is progressive. When *E*_2_ gains dynamical stability, the GOE is complete.

Another possibility is that the GOE is sudden. In this case, *E*_2_ gains dynamical stability first. An unstable interior fixed point, given by Eqs. (8), (9), (10), and (11), appears near *E*_2_ in phase space, and the interior fixed point moves toward *E*_1_ in phase space. When *E*_1_ loses dynamical stability, sudden oxygenation results, and the GOE is complete.

### Numerical integration

We used the fourth-order Runge-Kutta method to numerically integrate our differential equations.

### Reporting summary

Further information on research design is available in the Nature Research Reporting Summary linked to this article.

## Supplementary information

Supplementary Information

Reporting Summary

## Data Availability

All equations and parameter values are included in this article and in its SI file.

## Code availability

Code is available from the corresponding author upon reasonable request.
