# Supplementary material for: The Great Oxygenation Event as a consequence of ecological dynamics modulated by planetary change
Source: Nat Commun. 2021 Jun 28;12:3985. doi: 10.1038/s41467-021-23286-7 (PMC8238953; doi:10.1038/s41467-021-23286-7)
Supplement: Supplementary file 1 — Supplementary Information [file 41467_2021_23286_MOESM1_ESM.pdf]

Supplementary Information:

The Great Oxygenation Event as a consequence of ecological dynamics modulated by planetary change

Jason Olejarz[1], Yoh Iwasa[2], Andrew H. Knoll[1], Martin A. Nowak[1,3]

[1]Department of Organismic and Evolutionary Biology, Harvard University, Cambridge, MA 02138, USA
[2]Department of Bioscience, School of Science and Technology, Kwansei Gakuin University, Sanda-shi, Hyogo, Japan
[3]Department of Mathematics, Harvard University, Cambridge, MA 02138, USA

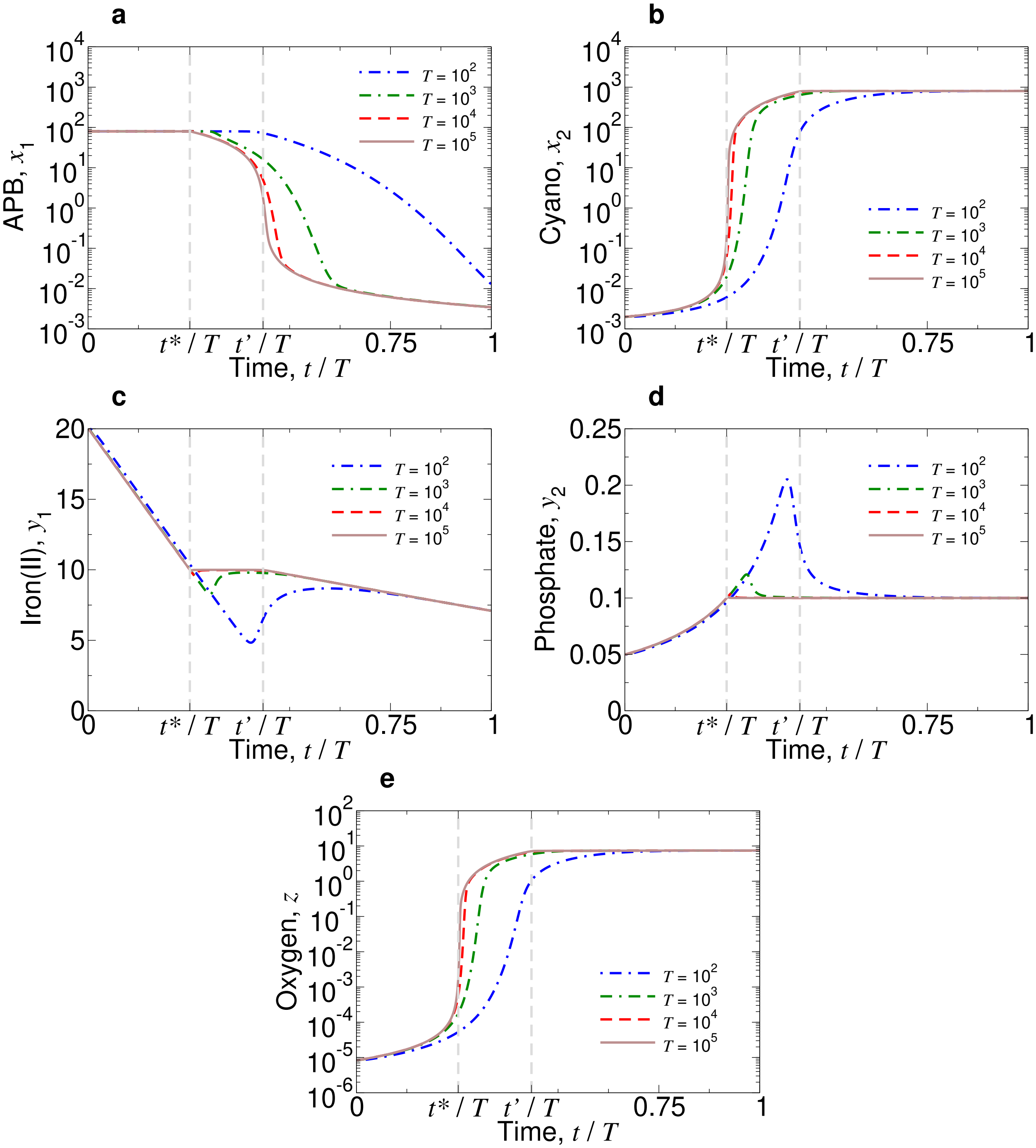

Fig. S1: **If the planet changes slowly relative to the timescale of bacterial reproduction and death, then at any given time, SI Equations** (8) **are approximately in steady state. A continuous transition is shown.** We simulate **SI** Equations (8) with $\alpha_1 = \alpha_2 = \beta_1 = \beta_2 = 1$ from time $t = 0$ until time $t = T$. We set $f_2 = 80$, $c = 10$, $a = 10$, $b = 100$, and $u_1 = u_2 = 10^{-3}$, and we set $f_1 = 100 - 40(t/T)$. t^* denotes the time at which Equilibrium E_1 loses stability in the limit $T \to \infty$, and t' denotes the time at which Equilibrium E_2 gains stability in the limit $T \to \infty$. We plot the abundances of APB (**a**), cyanobacteria (**b**), iron(II) (**c**), phosphate (**d**), and oxygen (**e**).

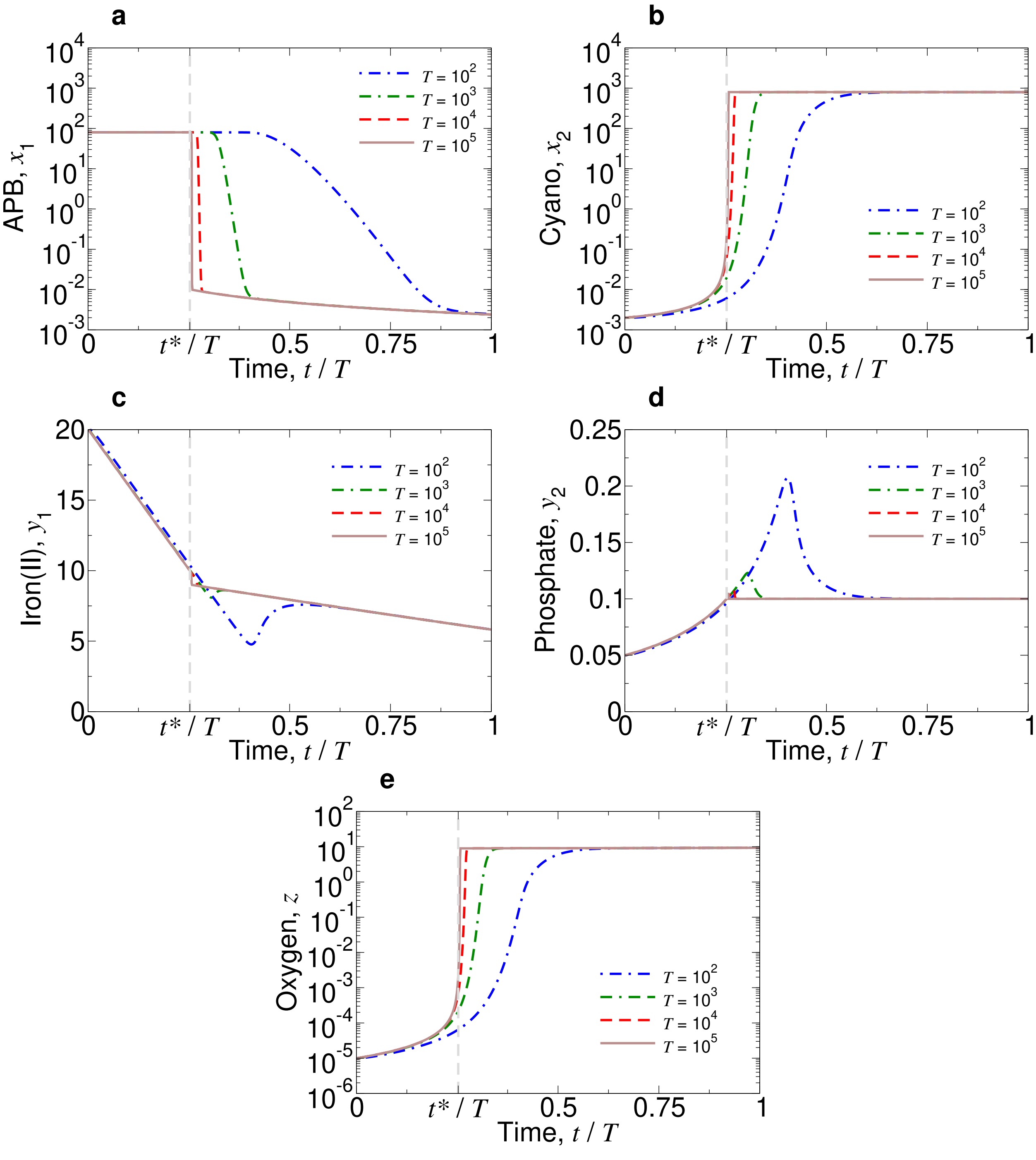

Fig. S2: **If the planet changes slowly relative to the timescale of bacterial reproduction and death, then at any given time, SI Equations** (8) **are approximately in steady state. A discontinuous transition is shown.** We simulate **SI** Equations (8) with $\alpha_1 = \alpha_2 = \beta_1 = \beta_2 = 1$ from time $t = 0$ until time $t = T$. We set $f_2 = 80$, $c = 10$, $a = 10$, $b = 80$, and $u_1 = u_2 = 10^{-3}$, and we set $f_1 = 100 - 40(t/T)$. t^* denotes the time at which a rapid transition occurs in the limit $T \to \infty$. We plot the abundances of APB (**a**), cyanobacteria (**b**), iron(II) (**c**), phosphate (**d**), and oxygen (**e**).

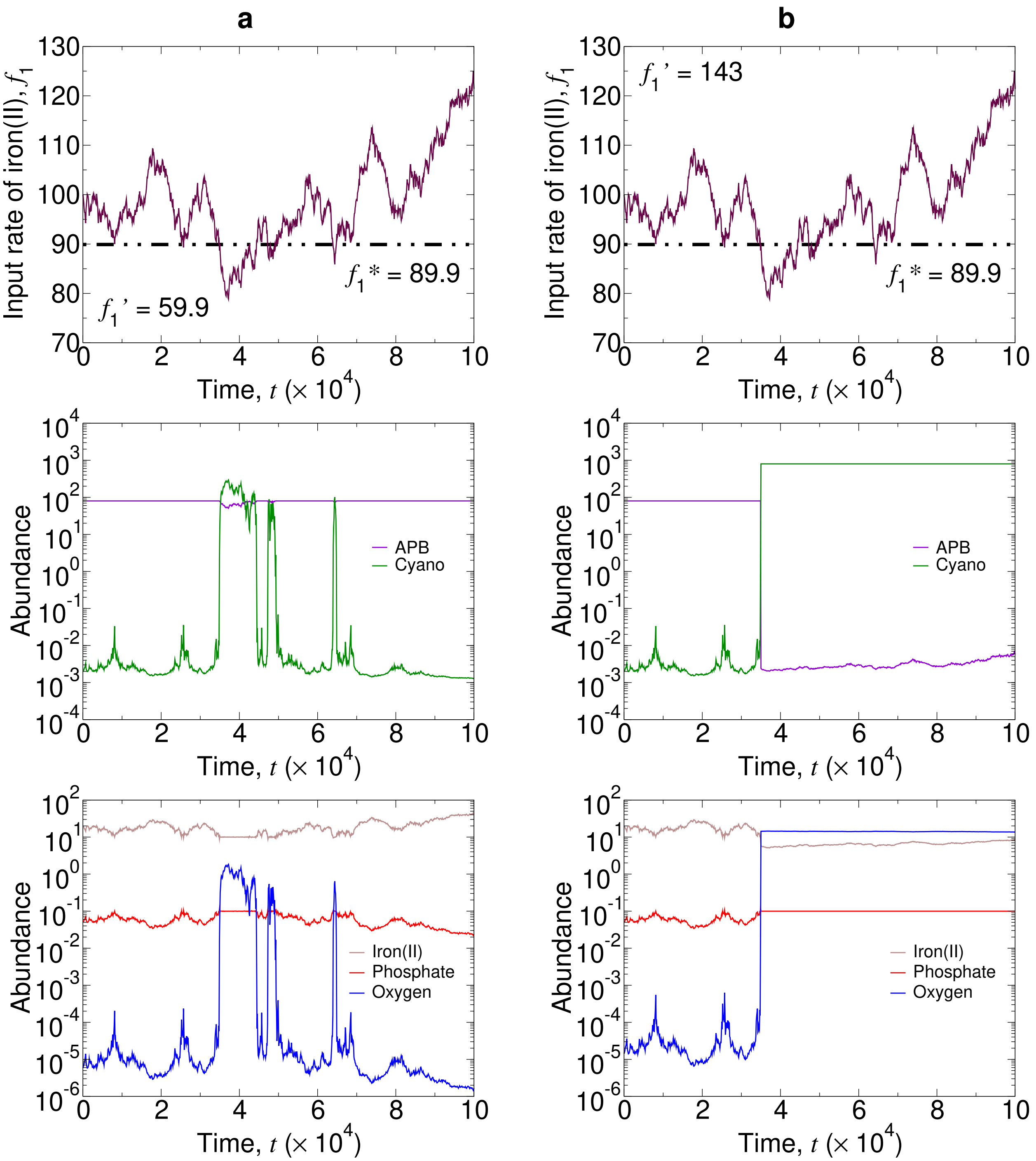

Fig. S3: **Whether or not oxygenation is reversible depends on the burial rate of oxygen.** Equilibrium E_1 (APB dominate) is stable for $f_1 > f_1^*$ and unstable for $f_1 < f_1^*$. We simulate **SI** Equations (8) with $\alpha_1 = \alpha_2 = \beta_1 = \beta_2 = 1$, and we set $f_2 = 80$, $c = 10$, $a = 10$, and $u_1 = u_2 = 10^{-3}$. **a**. For large values of b, oxygenation is readily reversed. We set $b = 150$. Since $f_1 > f_1'$, Equilibrium E_2 (cyanobacteria dominate) is unstable. **b**. For small values of b, oxygenation is robust to moderate amounts of geophysical change. We set $b = 50$. Since $f_1 < f_1'$, Equilibrium E_2 is stable.

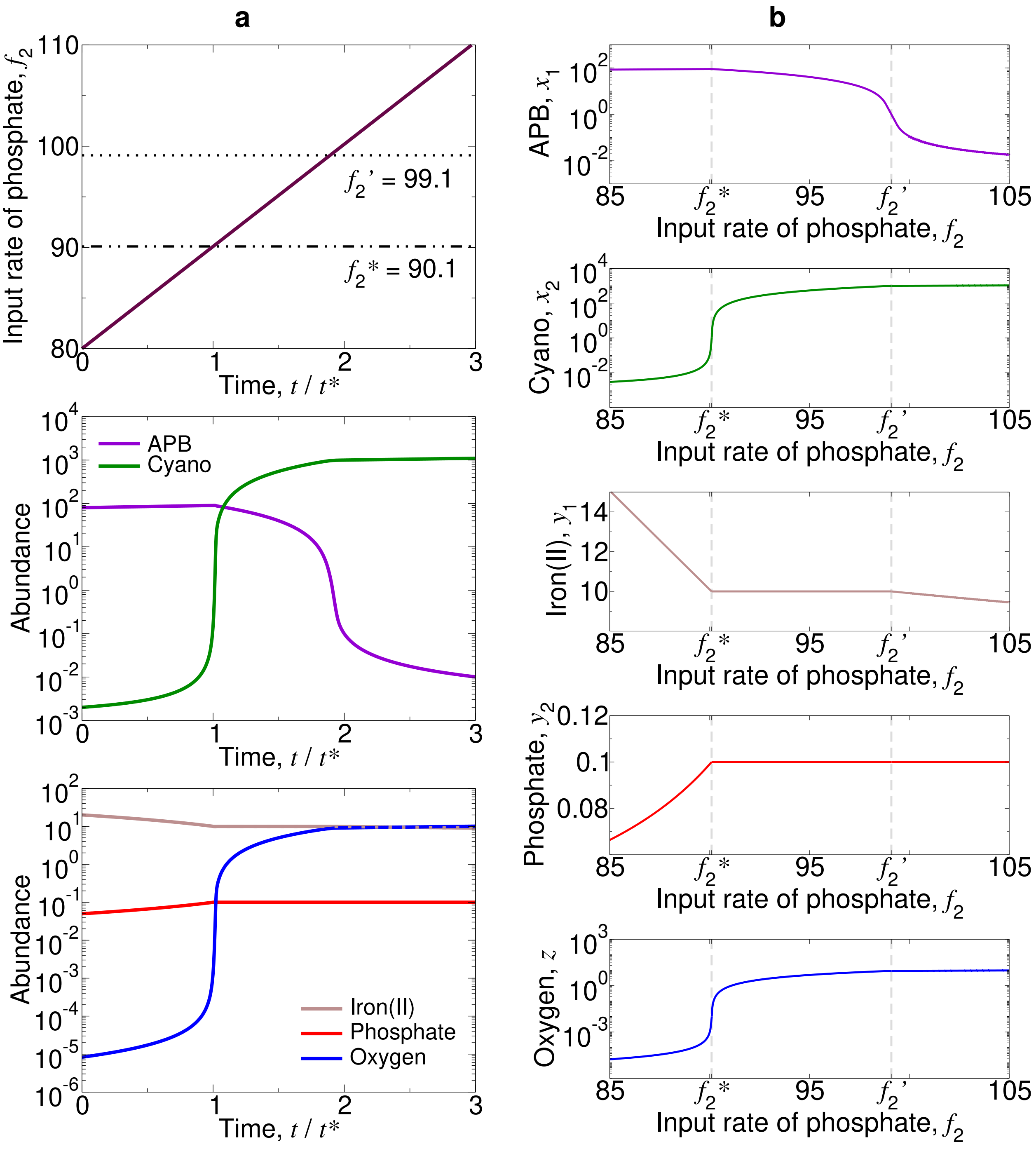

Fig. S4: **The GOE can be triggered by a rise in the influx of phosphate and is gradual if** $b > c(a-1)$**.** Equilibrium E_1 (APB dominate) loses stability and Equilibrium E_2 (cyanobacteria dominate) gains stability when f_2 rises above f_2^* and f_2', respectively. We set $f_1 = 100$, $c = 10$, $a = 10$, $b = 100$, and $u_1 = u_2 = 10^{-3}$. **a**. We simulate **SI** Equations (8) with $\alpha_1 = \alpha_2 = \beta_1 = \beta_2 = 1$, and we set $f_2 = 80 + 40(t/10^5)$. t^* denotes the time at which Equilibrium E_1 loses stability. **b**. There is stable coexistence of both types of bacteria for $f_2^* < f_2 < f_2'$.

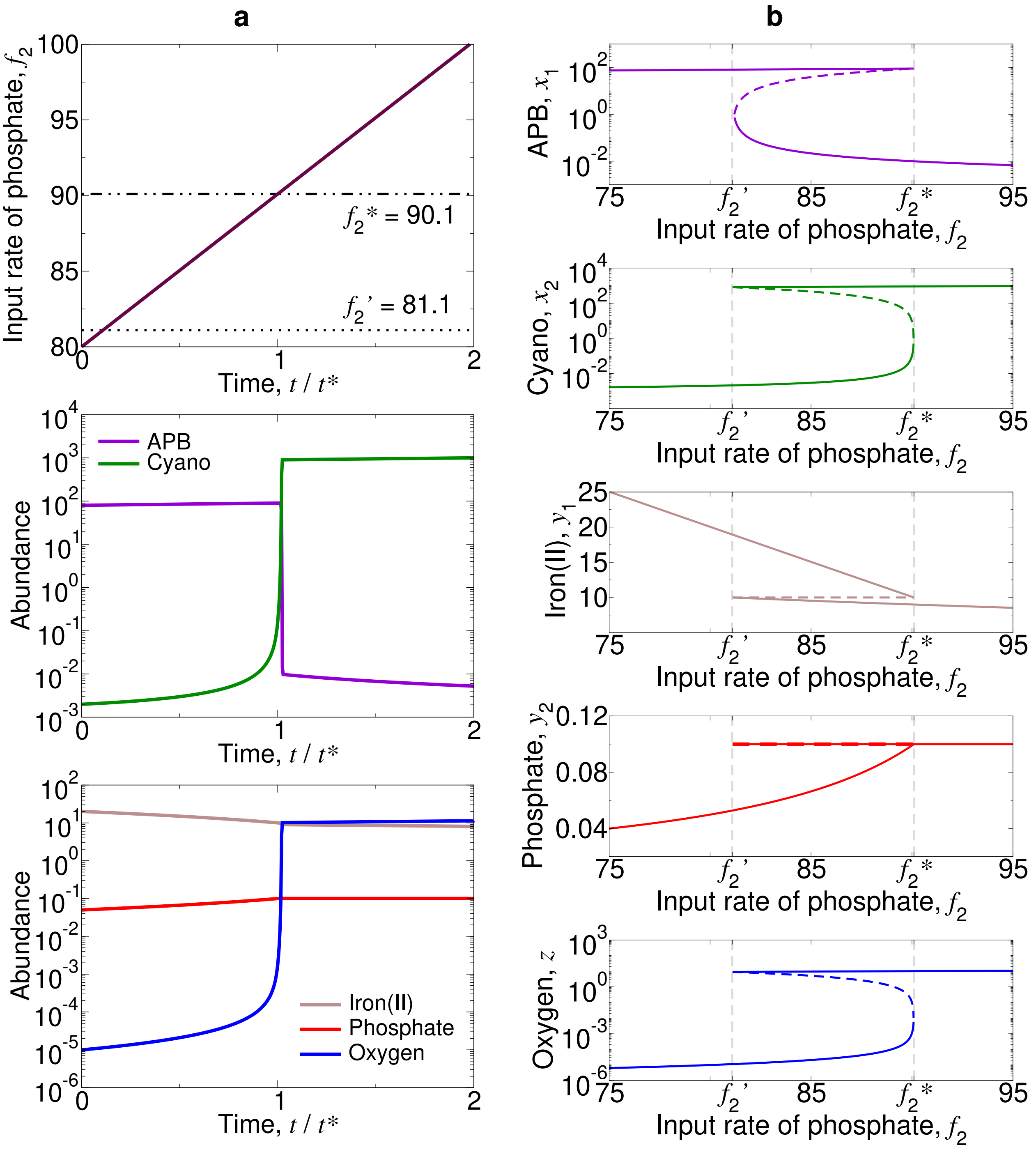

Fig. S5: **The GOE can be triggered by a rise in the influx of phosphate and is sudden if** $b < c(a-1)$**.** Equilibrium E_2 (cyanobacteria dominate) gains stability and Equilibrium E_1 (APB dominate) loses stability when f_2 rises above f_2' and f_2^*, respectively. We set $f_1 = 100$, $c = 10$, $a = 10$, $b = 80$, and $u_1 = u_2 = 10^{-3}$. **a**. We simulate **SI** Equations (8) with $\alpha_1 = \alpha_2 = \beta_1 = \beta_2 = 1$, and we set $f_2 = 80 + 40(t/10^5)$. t^* denotes the time at which Equilibrium E_1 loses stability. **b**. Bifurcation plots reveal bistability for $f_2' < f_2 < f_2^*$.

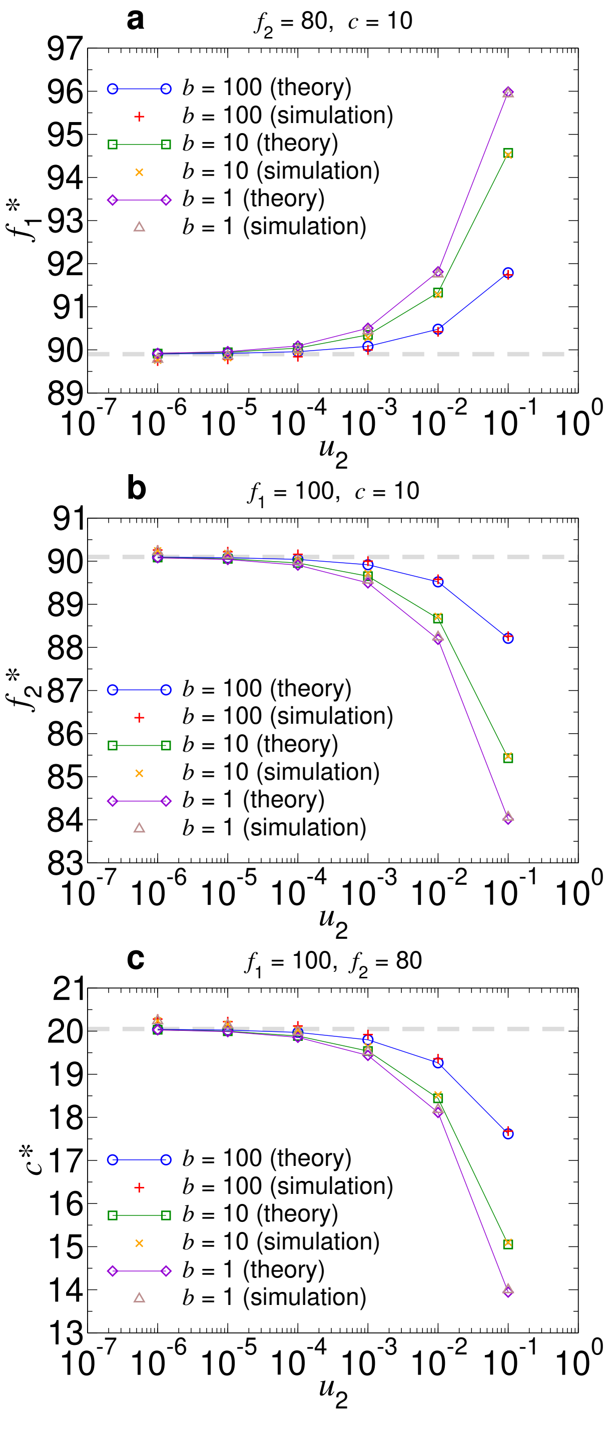

Fig. S6: **For small values of u_2, small values of a, and large values of b, the critical values of f_1, f_2, and c for triggering a GOE converge to the values given by SI Equations** (28)**,** (30)**, and** (32)**, respectively.** We set $a = 100$ and $u_1 = 10^{-3}$. We plot f_1^* (**a**), f_2^* (**b**), and c^* (**c**). The simulational values of f_1^*, f_2^*, and c^* are obtained from numerical integration of **SI** Equations (8) with $\alpha_1 = \alpha_2 = \beta_1 = \beta_2 = 1$. The theoretical values of f_1^*, f_2^*, and c^* are solutions to **SI** Equations (38), (39), and (40), respectively. The lines between the data points from **SI** Equations (38), (39), and (40) are a guide for the eye.

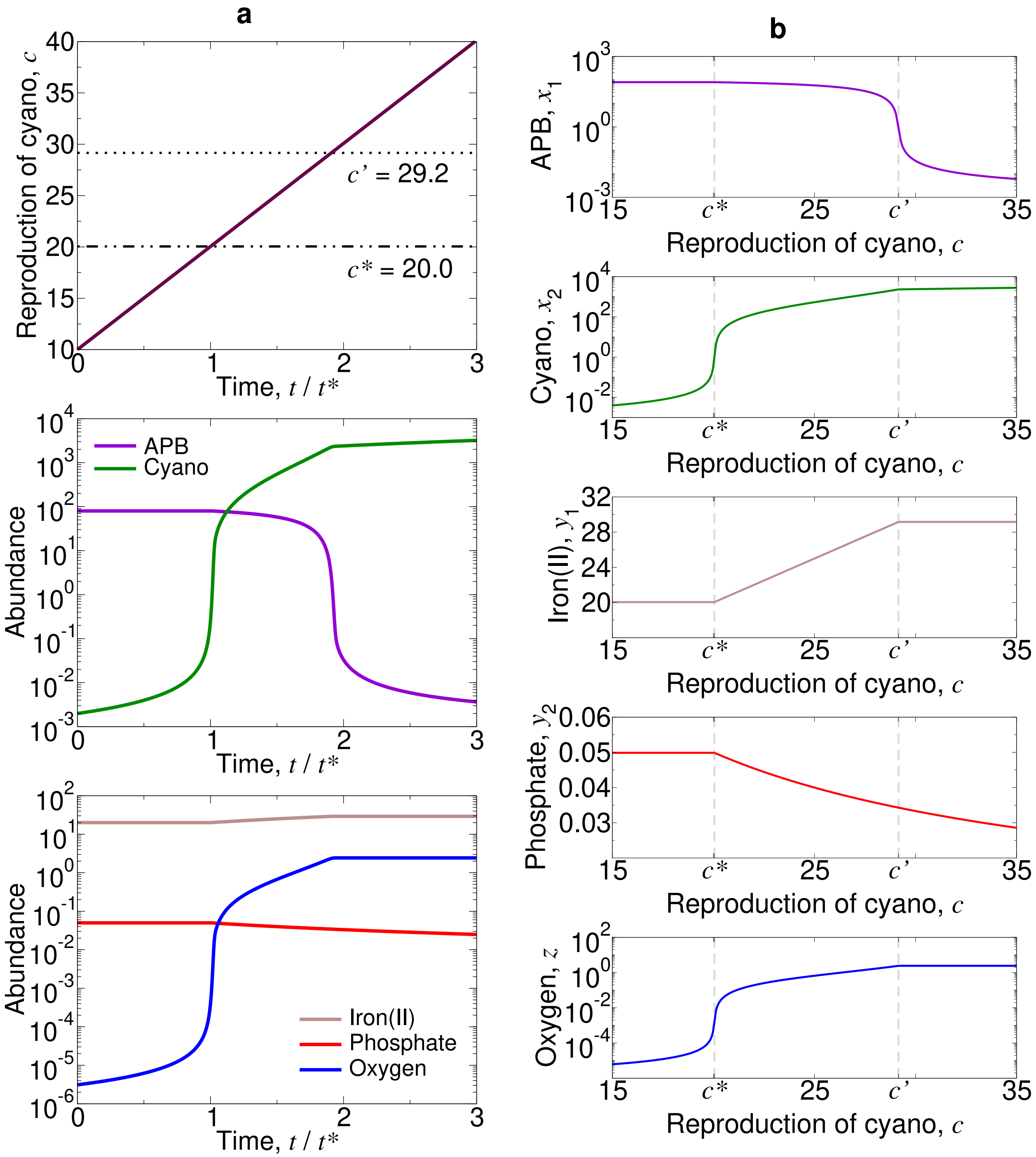

Fig. S7: **The GOE can be triggered by a rise in the reproductive rate of cyanobacteria and is gradual if** $b > c(a-1)$ **when** c **becomes greater than** c^***.** Equilibrium E_1 (APB dominate) loses stability and Equilibrium E_2 (cyanobacteria dominate) gains stability when c rises above c^* and c', respectively. We set $f_1 = 100$, $f_2 = 80$, $a = 10$, $b = 300$, and $u_1 = u_2 = 10^{-3}$. **a**. We simulate **SI** Equations (8) with $\alpha_1 = \alpha_2 = \beta_1 = \beta_2 = 1$, and we set $c = 10 + 40(t/10^5)$. t^* denotes the time at which Equilibrium E_1 loses stability. **b**. There is stable coexistence of both types of bacteria for $c^* < c < c'$.

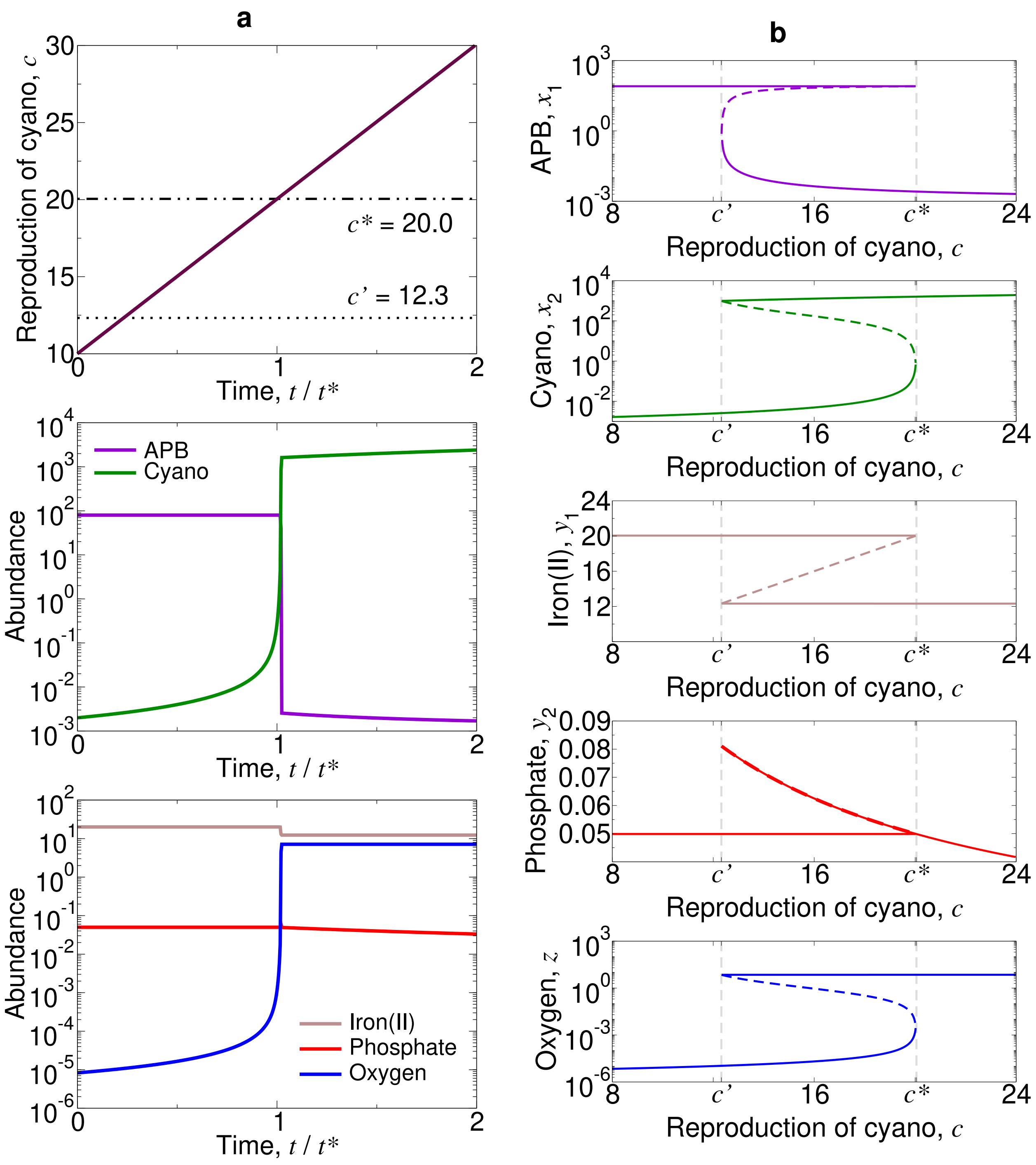

Fig. S8: **The GOE can be triggered by a rise in the reproductive rate of cyanobacteria and is sudden if $b < c(a-1)$ when c becomes greater than c^*.** Equilibrium E_2 (cyanobacteria dominate) gains stability and Equilibrium E_1 (APB dominate) loses stability when c rises above c' and c^*, respectively. We set $f_1 = 100$, $f_2 = 80$, $a = 10$, $b = 100$, and $u_1 = u_2 = 10^{-3}$. **a**. We simulate **SI** Equations (8) with $\alpha_1 = \alpha_2 = \beta_1 = \beta_2 = 1$, and we set $c = 10 + 40(t/10^5)$. t^* denotes the time at which Equilibrium E_1 loses stability. **b**. Bifurcation plots reveal bistability for $c' < c < c^*$.

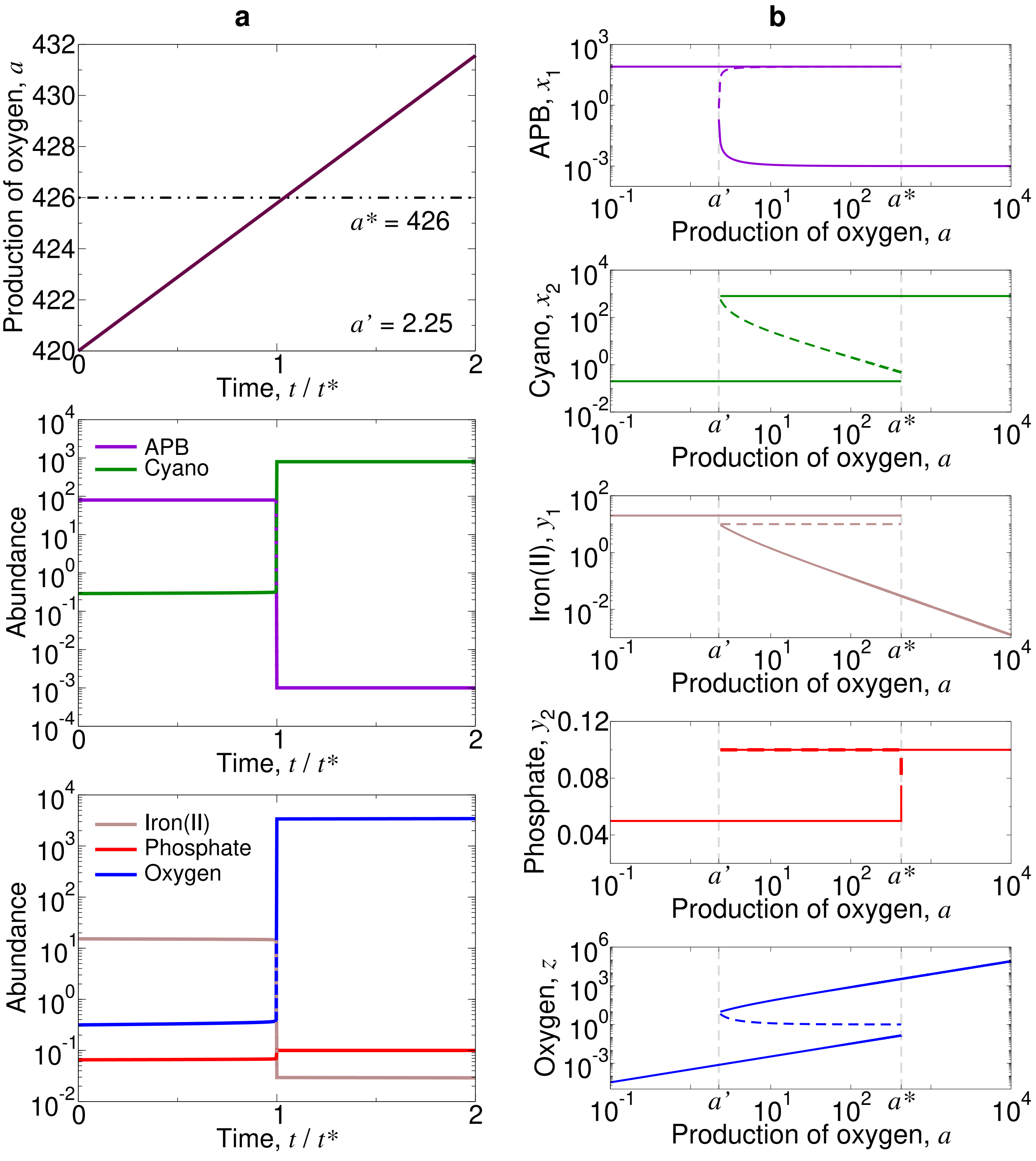

Fig. S9: **The GOE can be triggered by a rise in the production rate of oxygen.** Equilibrium E_2 (cyanobacteria dominate) gains stability and Equilibrium E_1 (APB dominate) ceases to exist when a rises above a' and a^*, respectively. We set $f_1 = 100$, $f_2 = 80$, $c = 10$, $b = 10$, $u_1 = 10^{-3}$, and $u_2 = 0.1$. **a**. We simulate **SI** Equations (8) with $\alpha_1 = \alpha_2 = \beta_1 = \beta_2 = 1$, and we set $a = 420 + 20(t/10^5)$. t^* denotes the time at which a saddle-node bifurcation occurs. **b**. Bifurcation plots reveal bistability for $a' < a < a^*$.

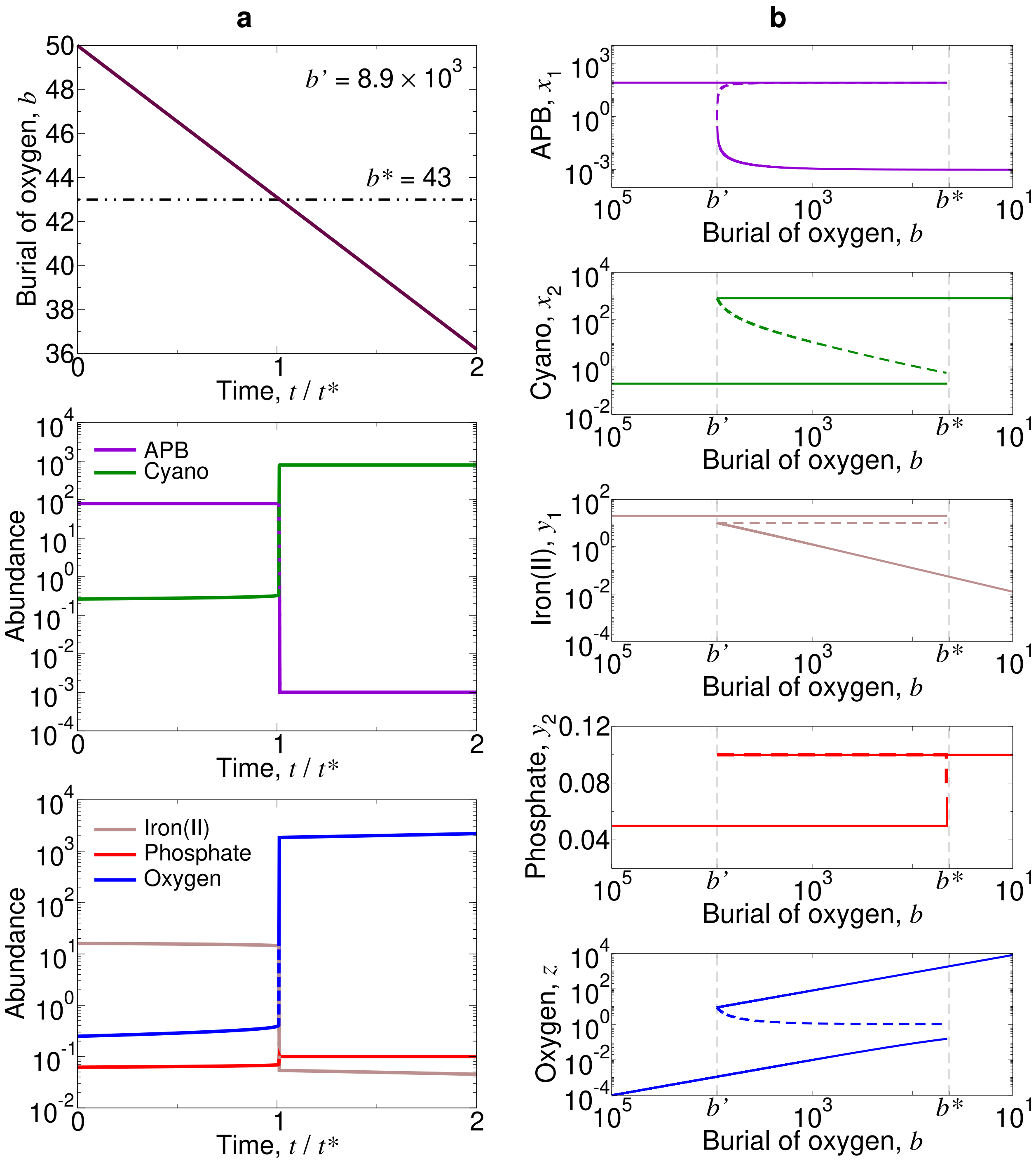

Fig. S10: **The GOE can be triggered by a decline in the burial rate of oxygen.** Equilibrium E_2 (cyanobacteria dominate) gains stability and Equilibrium E_1 (APB dominate) ceases to exist when b drops below b' and b^*, respectively. We set $f_1 = 100$, $f_2 = 80$, $c = 10$, $a = 10^3$, $u_1 = 10^{-3}$, and $u_2 = 0.1$. **a**. We simulate **SI** Equations (8) with $\alpha_1 = \alpha_2 = \beta_1 = \beta_2 = 1$, and we set $b = 50 - 20(t/10^5)$. t^* denotes the time at which a saddle-node bifurcation occurs. **b**. Bifurcation plots reveal bistability for $b^* < b < b'$.

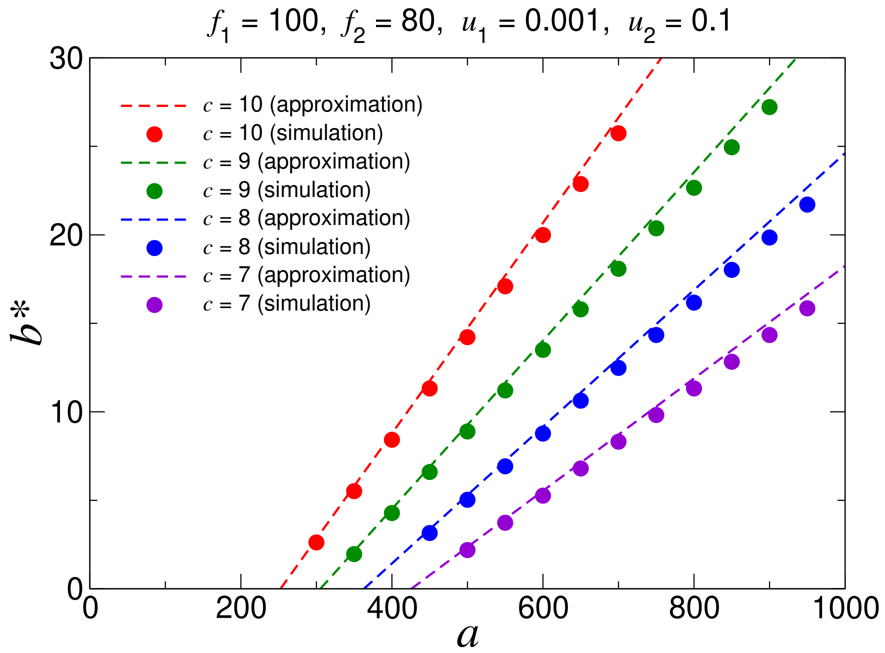

Fig. S11: **There is a critical value of a, given by SI Equation** (48)**, below which a declining value of b cannot trigger a GOE. SI** Equation (53) is plotted for $c = 10$, 9, 8, and 7 (dashed lines). The critical values of b are measured from numerical integration of **SI** Equations (8) with $\alpha_1 = \alpha_2 = \beta_1 = \beta_2 = 1$ (dots).

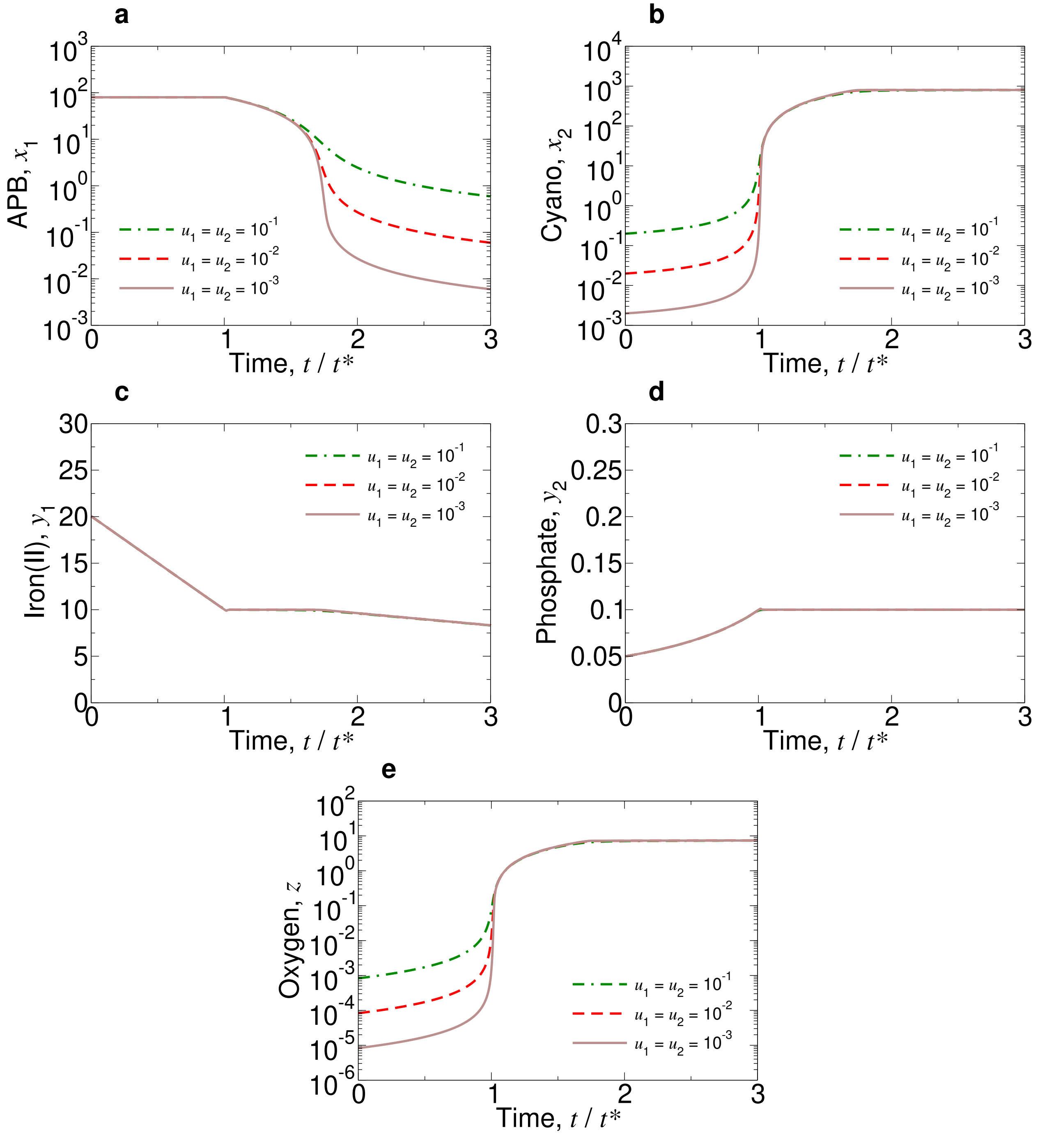

Fig. S12: **For larger values of the migration rates, the magnitudes of the changes in x_1, x_2, and z due to the GOE are smaller. A continuous transition is shown.** We set $f_2 = 80$, $c = 10$, $a = 10$, and $b = 100$. We simulate **SI** Equations (8) with $\alpha_1 = \alpha_2 = \beta_1 = \beta_2 = 1$, and we set $f_1 = 100 - 40(t/10^5)$. The transition is continuous. t^* denotes the time at which Equilibrium E_1 loses stability. We plot the abundances of APB (**a**), cyanobacteria (**b**), iron(II) (**c**), phosphate (**d**), and oxygen (**e**).

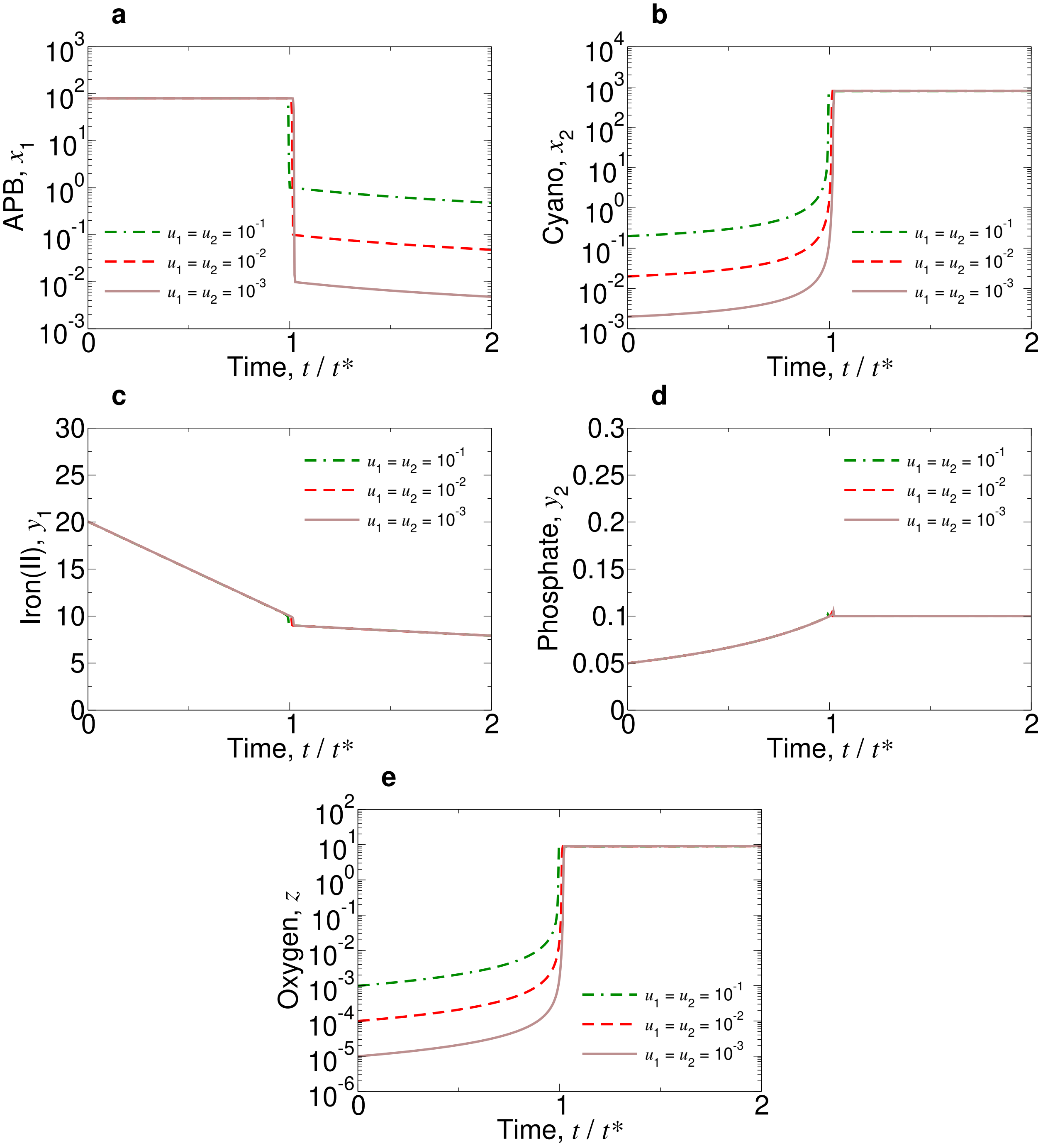

Fig. S13: **For larger values of the migration rates, the magnitudes of the changes in x_1, x_2, and z due to the GOE are smaller. A discontinuous transition is shown.** We set $f_2 = 80$, $c = 10$, $a = 10$, and $b = 80$. We simulate **SI** Equations (8) with $\alpha_1 = \alpha_2 = \beta_1 = \beta_2 = 1$, and we set $f_1 = 100 - 40(t/10^5)$. The transition is discontinuous. t^* denotes the time at which Equilibrium E_1 loses stability. We plot the abundances of APB (**a**), cyanobacteria (**b**), iron(II) (**c**), phosphate (**d**), and oxygen (**e**).

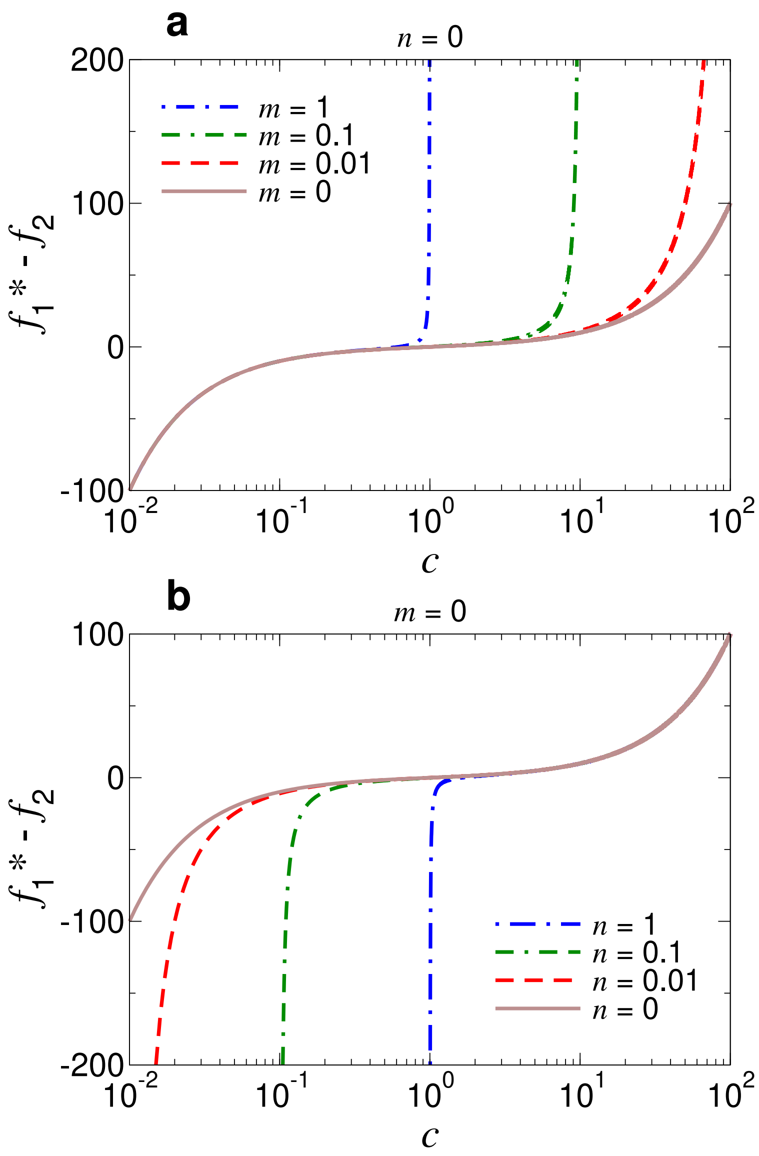

Fig. S14: **The condition for triggering a GOE depends on the parameters** m **and** n**.** In **SI** Equation (80), we set $f_1 = f_1^*$, and we set $p' = 0$. **a**. $f_1^* - f_2$ is plotted versus c for $n = 0$ and different values of m. **b**. $f_1^* - f_2$ is plotted versus c for $m = 0$ and different values of n.

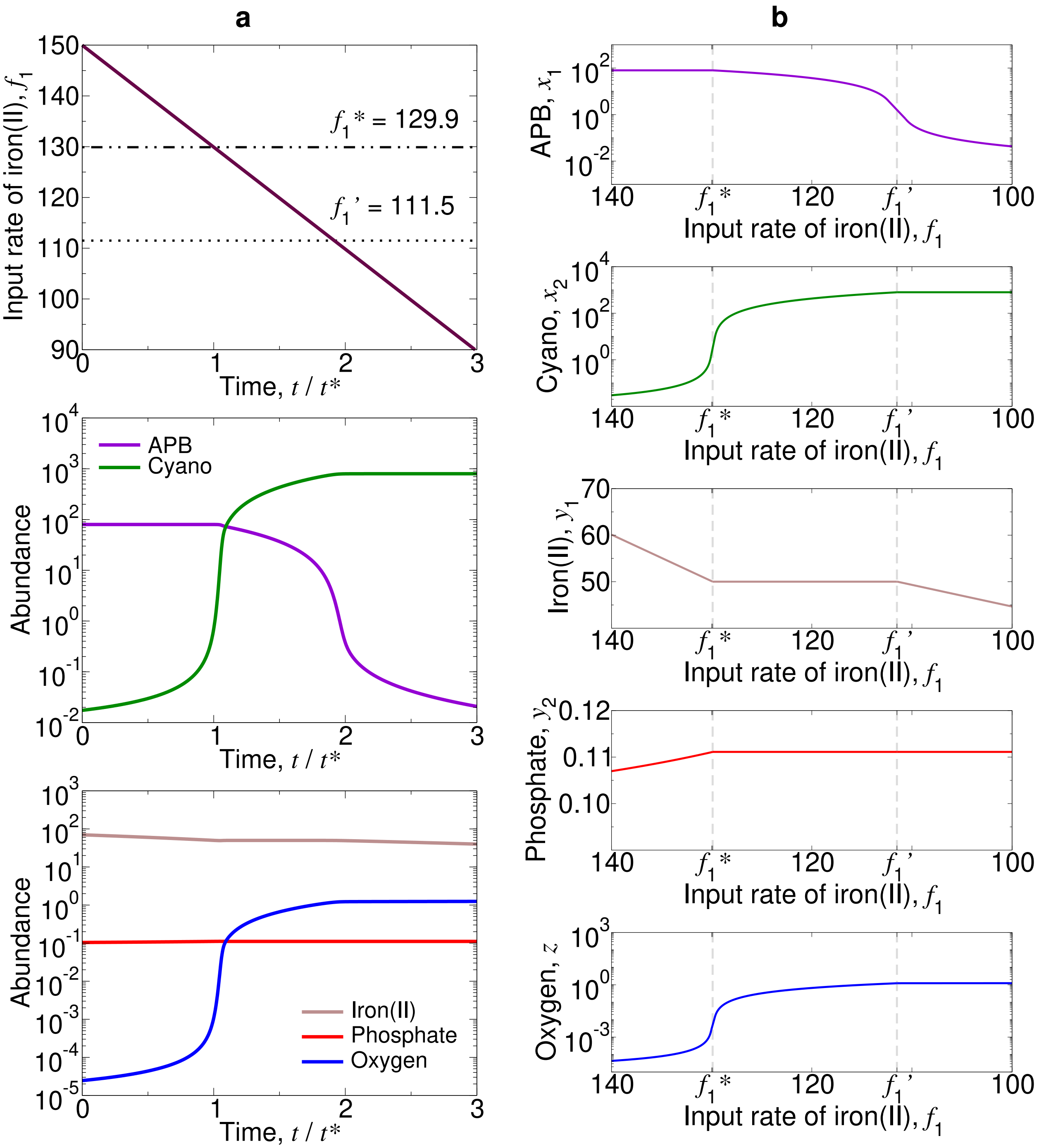

Fig. S15: **The GOE is gradual if** $b > c(a-1)/(1-mc)$**.** Equilibrium E_1 (APB dominate) loses stability and Equilibrium E_2 (cyanobacteria dominate) gains stability when f_1 drops below f_1^* and f_1', respectively. We set $m = 0.08$, $n = 1$, $f_2 = 80$, $c = 10$, $a = 10$, $b = 600$, and $u_1 = u_2 = 10^{-3}$. **a**. We simulate **SI** Equations (77) with $\alpha_1 = \alpha_2 = \beta_1 = \beta_2 = 1$, and we set $f_1 = 150 - 70(t/10^5)$. t^* denotes the time at which Equilibrium E_1 loses stability. **b**. There is stable coexistence of both types of bacteria for $f_1' < f_1 < f_1^*$.

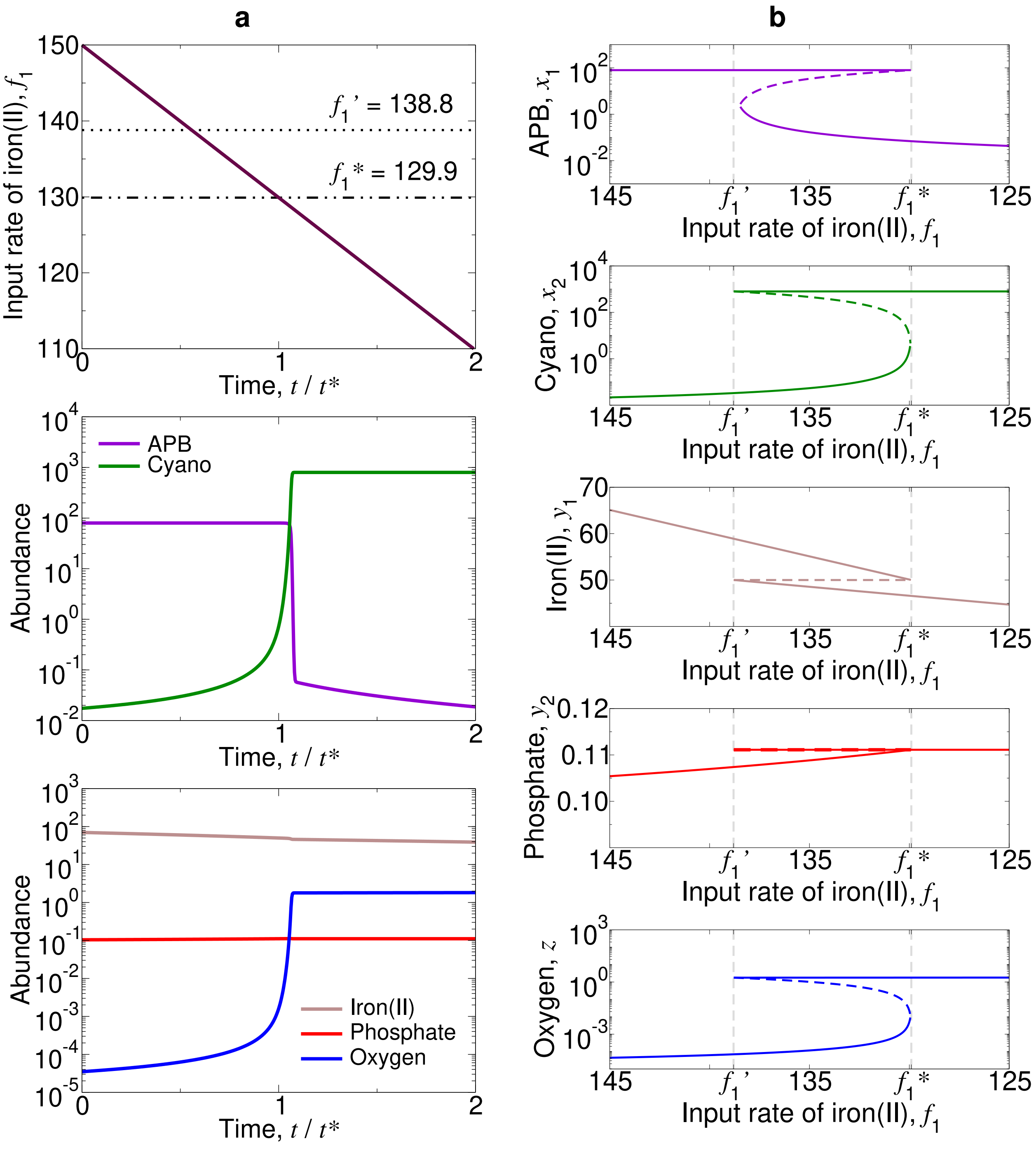

Fig. S16: **The GOE is sudden if** $b < c(a-1)/(1-mc)$**.** Equilibrium E_2 (cyanobacteria dominate) gains stability and Equilibrium E_1 (APB dominate) loses stability when f_1 drops below f_1' and f_1^*, respectively. We set $m = 0.08$, $n = 1$, $f_2 = 80$, $c = 10$, $a = 10$, $b = 400$, and $u_1 = u_2 = 10^{-3}$. **a**. We simulate **SI** Equations (77) with $\alpha_1 = \alpha_2 = \beta_1 = \beta_2 = 1$, and we set $f_1 = 150 - 70(t/10^5)$. t^* denotes the time at which Equilibrium E_1 loses stability. **b**. Bifurcation plots reveal bistability for $f_1^* < f_1 < f_1'$.

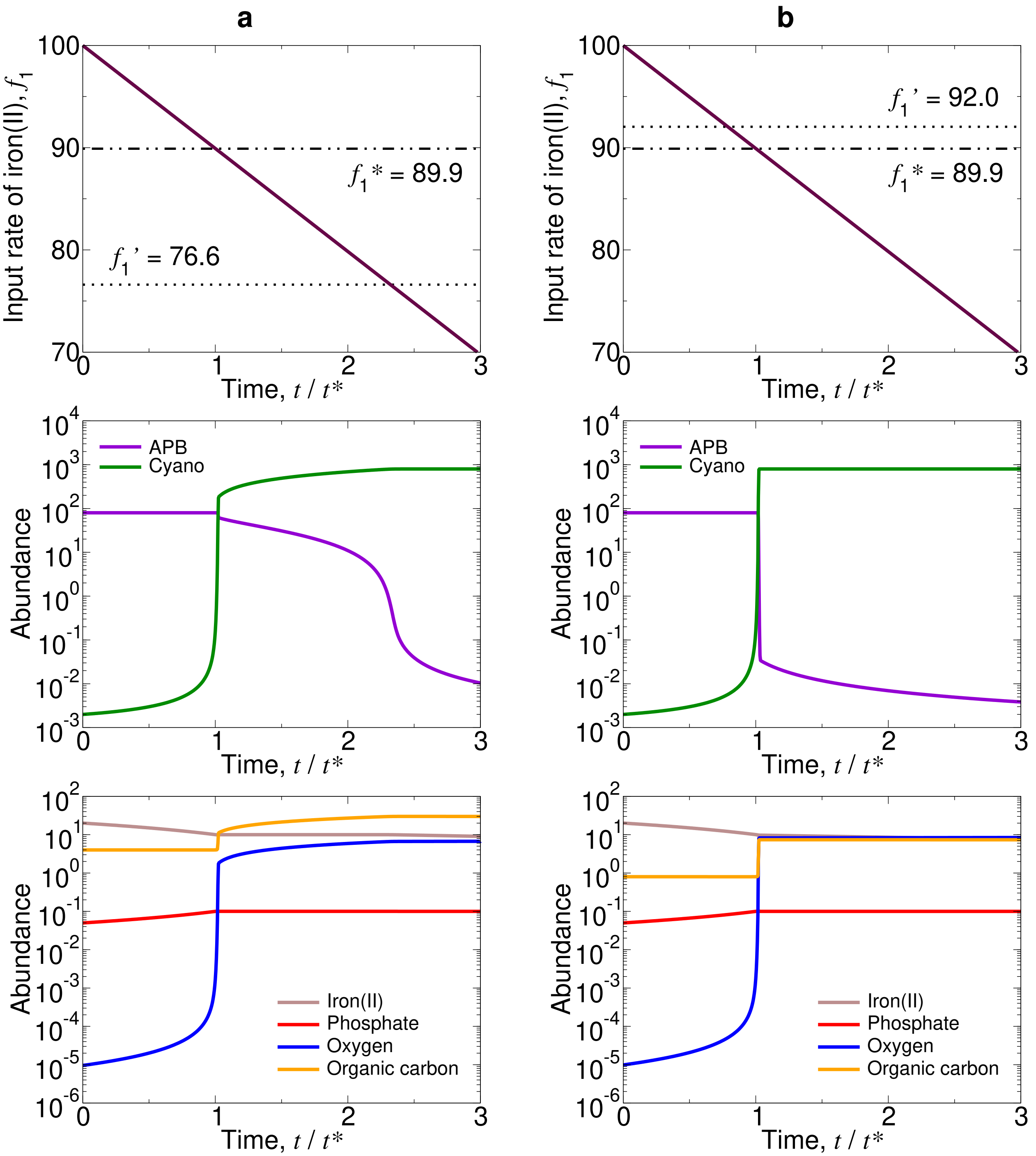

Fig. S17: **The rate of burial of organic carbon does not determine when the GOE is triggered, but it can affect the duration of the GOE.** We set $f_2 = 80$, $c = 10$, $a = 10$, $b = 80$, $j_1 = j_2 = 1$, and $u_1 = u_2 = 10^{-3}$. We simulate **SI** Equations (88) with $\alpha_1 = \alpha_2 = \beta_1 = \beta_2 = \Omega = 1$, and we set $f_1 = 100 - 40(t/10^5)$. **a**. We set $s = 20$. The GOE is gradual. **b**. We set $s = 100$. The GOE is sudden.

1 Supplementary Note 1: Ecological dynamics of anoxygenic photosynthetic bacteria and cyanobacteria

For understanding the mechanisms behind the GOE, we investigate the following system of ordinary differential equations:

$$\begin{aligned}
\frac{dX_1}{dT} &= C_1X_1Y_1Y_2 - D_1X_1 + U_1 \\
\frac{dX_2}{dT} &= C_2X_2Y_2 - D_2X_2 + U_2 \\
\frac{dY_1}{dT} &= F_1 - G_1Y_1 - HX_1Y_1Y_2 - R_1Y_1Z \\
\frac{dY_2}{dT} &= F_2 - G_2Y_2 - H_1X_1Y_1Y_2 - H_2X_2Y_2 \\
\frac{dZ}{dT} &= AX_2Y_2 - BZ - RY_1Z
\end{aligned} \tag{1}$$

X_1 is the abundance of anoxygenic photosynthetic bacteria (APB), X_2 is the abundance of cyanobacteria, Y_1 is the abundance of iron(II) (Fe^{2+}), Y_2 is the abundance of phosphate (${PO_4}^{3-}$), and Z is the abundance of dioxygen (O_2).

All parameters of the model are necessarily positive. APB proliferate at rate $C_1X_1Y_1Y_2$, while cyanobacteria proliferate at rate $C_2X_2Y_2$. APB die at per-capita rate D_1, while cyanobacteria die at per-capita rate D_2. APB migrate into the system at rate U_1, while cyanobacteria migrate into the system at rate U_2. Setting $U_1 > 0$ and $U_2 > 0$ guarantees that there is always competition between the two types of bacteria.

Iron(II) is input at rate F_1 and lost inorganically at rate G_1Y_1. APB use iron(II) for reproduction, so iron(II) is lost organically at rate $HX_1Y_1Y_2$. Dioxygen rusts iron, reducing the amount of iron at rate R_1Y_1Z. Phosphate is input at rate F_2 and lost inorganically at rate G_2Y_2. APB and cyanobactera both use phosphate for reproduction, so phosphate is also lost organically at rates $H_1X_1Y_1Y_2$ and $H_2X_2Y_2$. Dioxygen is produced by cyanobacteria and enters the system at rate AX_2Y_2. Dioxygen is buried at rate BZ and lost due to rusting at rate RY_1Z.

1.1 Rescaling

For understanding the ecological dynamics and fixed points of Equations (1), we can reduce the size of the parameter space without loss of generality. Equations (1) can be rewritten as

$$\begin{aligned}
\frac{d}{dT}(HX_1) &= (HX_1)(C_1Y_1)Y_2 - D_1(HX_1) + HU_1 \\
\frac{d}{dT}(H_2X_2) &= C_2(H_2X_2)Y_2 - D_2(H_2X_2) + H_2U_2 \\
\frac{d}{dT}(C_1Y_1) &= C_1F_1 - G_1(C_1Y_1) - (HX_1)(C_1Y_1)Y_2 - (C_1Y_1)(R_1Z) \\
\frac{dY_2}{dT} &= F_2 - G_2Y_2 - \left(\frac{H_1}{C_1H}\right)(HX_1)(C_1Y_1)Y_2 - (H_2X_2)Y_2 \\
\frac{d}{dT}(R_1Z) &= \left(\frac{AR_1}{H_2}\right)(H_2X_2)Y_2 - B(R_1Z) - \left(\frac{R}{C_1}\right)(C_1Y_1)(R_1Z)
\end{aligned} \tag{2}$$

We can further rewrite Equations (2) as

$$\begin{aligned}
\left(\frac{1}{D_1}\right)\frac{d}{dT}\left(\frac{HX_1}{G_1}\right) &= \left(\frac{HX_1}{G_1}\right)\left(\frac{C_1Y_1}{D_1}\right)Y_2 - \frac{HX_1}{G_1} + \frac{HU_1}{D_1G_1} \\
\left(\frac{1}{D_2}\right)\frac{d}{dT}\left(\frac{H_2X_2}{G_2}\right) &= \left(\frac{C_2}{D_2}\right)\left(\frac{H_2X_2}{G_2}\right)Y_2 - \frac{H_2X_2}{G_2} + \frac{H_2U_2}{D_2G_2} \\
\left(\frac{1}{G_1}\right)\frac{d}{dT}\left(\frac{C_1Y_1}{D_1}\right) &= \frac{C_1F_1}{D_1G_1} - \frac{C_1Y_1}{D_1} \\
&\quad - \left(\frac{HX_1}{G_1}\right)\left(\frac{C_1Y_1}{D_1}\right)Y_2 - \left(\frac{C_1Y_1}{D_1}\right)\left(\frac{R_1Z}{G_1}\right) \\
\left(\frac{1}{G_2}\right)\frac{dY_2}{dT} &= \frac{F_2}{G_2} - Y_2 \\
&\quad - \left(\frac{D_1G_1H_1}{C_1G_2H}\right)\left(\frac{HX_1}{G_1}\right)\left(\frac{C_1Y_1}{D_1}\right)Y_2 - \left(\frac{H_2X_2}{G_2}\right)Y_2 \\
\left(\frac{C_1}{D_1R}\right)\frac{d}{dT}\left(\frac{R_1Z}{G_1}\right) &= \left(\frac{AC_1G_2R_1}{D_1G_1H_2R}\right)\left(\frac{H_2X_2}{G_2}\right)Y_2 \\
&\quad - \left(\frac{BC_1}{D_1R}\right)\left(\frac{R_1Z}{G_1}\right) - \left(\frac{C_1Y_1}{D_1}\right)\left(\frac{R_1Z}{G_1}\right)
\end{aligned} \tag{3}$$

We define

$$k \equiv \sqrt{\frac{D_1G_1H_1}{C_1G_2H}} \tag{4}$$

Using Equation (4), Equations (3) can be rewritten as

$$\begin{aligned}
\left(\frac{1}{D_1}\right)\frac{d}{dT}\left(\frac{kHX_1}{G_1}\right) &= \left(\frac{kHX_1}{G_1}\right)\left(\frac{kC_1Y_1}{D_1}\right)\left(\frac{Y_2}{k}\right) - \frac{kHX_1}{G_1} + \frac{kHU_1}{D_1G_1} \\
\left(\frac{1}{D_2}\right)\frac{d}{dT}\left(\frac{H_2X_2}{G_2}\right) &= \left(\frac{kC_2}{D_2}\right)\left(\frac{H_2X_2}{G_2}\right)\left(\frac{Y_2}{k}\right) - \frac{H_2X_2}{G_2} + \frac{H_2U_2}{D_2G_2} \\
\left(\frac{1}{G_1}\right)\frac{d}{dT}\left(\frac{kC_1Y_1}{D_1}\right) &= \frac{kC_1F_1}{D_1G_1} - \frac{kC_1Y_1}{D_1} \\
&\quad - \left(\frac{kHX_1}{G_1}\right)\left(\frac{kC_1Y_1}{D_1}\right)\left(\frac{Y_2}{k}\right) - \left(\frac{kC_1Y_1}{D_1}\right)\left(\frac{R_1Z}{G_1}\right) \\
\left(\frac{1}{G_2}\right)\frac{d}{dT}\left(\frac{Y_2}{k}\right) &= \frac{F_2}{kG_2} - \frac{Y_2}{k} \\
&\quad - \left(\frac{kHX_1}{G_1}\right)\left(\frac{kC_1Y_1}{D_1}\right)\left(\frac{Y_2}{k}\right) - \left(\frac{H_2X_2}{G_2}\right)\left(\frac{Y_2}{k}\right) \\
\left(\frac{kC_1}{D_1R}\right)\frac{d}{dT}\left(\frac{R_1Z}{G_1}\right) &= \left(\frac{k^2AC_1G_2R_1}{D_1G_1H_2R}\right)\left(\frac{H_2X_2}{G_2}\right)\left(\frac{Y_2}{k}\right) \\
&\quad - \left(\frac{kBC_1}{D_1R}\right)\left(\frac{R_1Z}{G_1}\right) - \left(\frac{kC_1Y_1}{D_1}\right)\left(\frac{R_1Z}{G_1}\right)
\end{aligned} \tag{5}$$

To simplify Equations (5), we make the following definitions:

$$
\begin{aligned}
a &\equiv \frac{k^2 A C_1 G_2 R_1}{D_1 G_1 H_2 R} \\
b &\equiv \frac{k B C_1}{D_1 R} \\
c &\equiv \frac{k C_2}{D_2} \\
f_1 &\equiv \frac{k C_1 F_1}{D_1 G_1} \\
f_2 &\equiv \frac{F_2}{k G_2} \\
u_1 &\equiv \frac{k H U_1}{D_1 G_1} \\
u_2 &\equiv \frac{H_2 U_2}{D_2 G_2} \\
x_1 &\equiv \frac{k H X_1}{G_1} \\
x_2 &\equiv \frac{H_2 X_2}{G_2} \\
y_1 &\equiv \frac{k C_1 Y_1}{D_1} \\
y_2 &\equiv \frac{Y_2}{k} \\
z &\equiv \frac{R_1 Z}{G_1}
\end{aligned}
\tag{6}
$$

We also define

$$
\begin{aligned}
\gamma &\equiv \frac{k C_1}{D_1 R} \\
t &\equiv \frac{T}{\gamma} \\
\alpha_1 &\equiv \frac{1}{\gamma D_1} \\
\alpha_2 &\equiv \frac{1}{\gamma D_2} \\
\beta_1 &\equiv \frac{1}{\gamma G_1} \\
\beta_2 &\equiv \frac{1}{\gamma G_2}
\end{aligned}
\tag{7}
$$

Substituting Equations (6) and (7) into Equations (5), we obtain

$$\begin{aligned}
\alpha_1\dot{x}_1 &= x_1y_1y_2 - x_1 + u_1\\
\alpha_2\dot{x}_2 &= cx_2y_2 - x_2 + u_2\\
\beta_1\dot{y}_1 &= f_1 - y_1 - x_1y_1y_2 - y_1z\\
\beta_2\dot{y}_2 &= f_2 - y_2 - x_1y_1y_2 - x_2y_2\\
\dot{z} &= ax_2y_2 - bz - y_1z
\end{aligned} \tag{8}$$

An overdot denotes the derivative with respect to t. For studying the ecological dynamics, without loss of generality, we consider Equations (8).

1.1.1 Two-dimensional approximation of the dynamics

From Equations (8), for approximating the dynamics, we can also consider a simplified system of equations:

$$\begin{aligned}
\alpha_1\dot{x}_1 &\approx x_1y_1y_2 - x_1 + u_1\\
\alpha_2\dot{x}_2 &\approx cx_2y_2 - x_2 + u_2\\
0 &\approx f_1 - y_1 - x_1y_1y_2 - y_1z\\
0 &\approx f_2 - y_2 - x_1y_1y_2 - x_2y_2\\
0 &\approx ax_2 - bz
\end{aligned} \tag{9}$$

We define

$$\xi \equiv \frac{b}{a} \tag{10}$$

Using Equation (10), from the fifth of Equations (9), we have

$$z \approx \frac{x_2}{\xi} \tag{11}$$

Using Equation (11), from the third and fourth of Equations (9), we have

$$\begin{aligned}
y_1 \approx \frac{1}{2x_1(\xi + x_2)}\Big[&- (1 + x_2)(\xi + x_2) + \xi x_1(f_1 - f_2)\\
&+ \sqrt{[(1 + x_2)(\xi + x_2) - \xi x_1(f_1 - f_2)]^2 + 4\xi f_1x_1(1 + x_2)(\xi + x_2)}\Big]
\end{aligned} \tag{12}$$

We also have

$$\begin{aligned}
y_2 \approx \frac{1}{2\xi x_1(1 + x_2)}\Big[&- (1 + x_2)(\xi + x_2) - \xi x_1(f_1 - f_2)\\
&+ \sqrt{[(1 + x_2)(\xi + x_2) - \xi x_1(f_1 - f_2)]^2 + 4\xi f_1x_1(1 + x_2)(\xi + x_2)}\Big]
\end{aligned} \tag{13}$$

Substituting Equations (12) and (13) into the first and second of Equations (9), we obtain

$$\begin{aligned}
\alpha_1 \dot{x}_1 \approx & \frac{x_1}{4\xi x_1^2(1+x_2)(\xi+x_2)} \Bigg[-(1+x_2)(\xi+x_2)+\xi x_1(f_1-f_2) \\
& + \sqrt{[(1+x_2)(\xi+x_2)-\xi x_1(f_1-f_2)]^2+4\xi f_1 x_1(1+x_2)(\xi+x_2)} \Bigg] \\
& \times \Bigg[-(1+x_2)(\xi+x_2)-\xi x_1(f_1-f_2) \\
& + \sqrt{[(1+x_2)(\xi+x_2)-\xi x_1(f_1-f_2)]^2+4\xi f_1 x_1(1+x_2)(\xi+x_2)} \Bigg] - x_1 + u_1 \\
\alpha_2 \dot{x}_2 \approx & \frac{c x_2}{2\xi x_1(1+x_2)} \Bigg[-(1+x_2)(\xi+x_2)-\xi x_1(f_1-f_2) \\
& + \sqrt{[(1+x_2)(\xi+x_2)-\xi x_1(f_1-f_2)]^2+4\xi f_1 x_1(1+x_2)(\xi+x_2)} \Bigg] - x_2 + u_2
\end{aligned} \tag{14}$$

For constructing phase portraits that approximately represent the dynamics of x_1 and x_2, we use Equations (14).

1.2 Fixed points

The fixed points of the ecological dynamics are obtained from the steady state of Equations (8):

$$\begin{aligned}
0 &= \bar{\bar{x}}_1\bar{\bar{y}}_1\bar{\bar{y}}_2 - \bar{\bar{x}}_1 + u_1 \\
0 &= c\bar{\bar{x}}_2\bar{\bar{y}}_2 - \bar{\bar{x}}_2 + u_2 \\
0 &= f_1 - \bar{\bar{y}}_1 - \bar{\bar{x}}_1\bar{\bar{y}}_1\bar{\bar{y}}_2 - \bar{\bar{y}}_1\bar{\bar{z}} \\
0 &= f_2 - \bar{\bar{y}}_2 - \bar{\bar{x}}_1\bar{\bar{y}}_1\bar{\bar{y}}_2 - \bar{\bar{x}}_2\bar{\bar{y}}_2 \\
0 &= a\bar{\bar{x}}_2\bar{\bar{y}}_2 - b\bar{\bar{z}} - \bar{\bar{y}}_1\bar{\bar{z}}
\end{aligned} \tag{15}$$

We solve for the fixed points of Equations (8) without loss of generality by considering Equations (15).

1.3 Basic considerations

We assume that on the time scale of reproduction and death of the bacteria, the parameters in Equations (8) are approximately constant. This means that at any given time, x_1, x_2, y_1, y_2, and z in Equations (8) are approximately in steady state. On a much longer time scale, as the planet slowly changes its physical and chemical makeup, the parameters in Equations (8) change accordingly. This separation of time scales between that of planetary change and that of the ecological dynamics of the bacteria means that, for understanding the GOE, we can focus on the fixed points of Equations (8)—given by Equations (15)—and their dynamical stability (**Fig. S1** and **Fig. S2**). The key consideration then becomes how the fixed points given by Equations (15) and their stability properties depend on the parameters of the model.

The foundation of our understanding of the GOE is based on assuming that the migration rates, u_1 and u_2, are small. Setting $u_1 = u_2 = 0$ in Equations (8), there are a few basic considerations:

- **Can the fixed point for which cyanobacteria are extinct be invaded by a small amount of cyanobacteria?** Prior to the GOE, a small amount of cyanobacteria cannot grow to establish a stable population of cyanobacteria. As parameter values change on geological time scales, however, conditions can become favorable for cyanobacteria to outcompete APB.
- **Is there a fixed point for which APB and cyanobacteria stably coexist?** If there is, then oxygenation of Earth's atmosphere can be gradual. If there is not, then oxygenation can be sudden and drastic.
- **Can the fixed point for which APB are extinct be invaded by a small amount of APB?** If it cannot, then after the GOE, the state of the global ecosystem is robust—parameter values might change in the reverse direction, but cyanobacteria remain dominant. This can result in hysteresis in the chemical makeup of Earth's atmosphere.

1.4 Mathematical analysis with arbitrarily small migration

u_1 and u_2 are always positive, but for understanding the causes of the GOE, it is helpful to assume that they are arbitrarily small. Accordingly, we characterize the case where $u_1 = u_2 = 0$. We obtain simple analytical results for the fixed points and their stability properties.

1.4.1 Fixed points

We begin by calculating the fixed points of Equations (8) and their stability properties for the case of no migration. Setting $u_1 = u_2 = 0$ in Equations (15), we have

$$\begin{aligned}
0 &= (\bar{y}_1\bar{y}_2 - 1)\bar{x}_1 \\
0 &= (c\bar{y}_2 - 1)\bar{x}_2 \\
0 &= f_1 - \bar{y}_1 - \bar{x}_1\bar{y}_1\bar{y}_2 - \bar{y}_1\bar{z} \\
0 &= f_2 - \bar{y}_2 - \bar{x}_1\bar{y}_1\bar{y}_2 - \bar{x}_2\bar{y}_2 \\
0 &= a\bar{x}_2\bar{y}_2 - b\bar{z} - \bar{y}_1\bar{z}
\end{aligned} \tag{16}$$

We set $\bar{x}_2 = 0$ with $\bar{x}_1 > 0$ in Equations (16) to calculate the fixed point for which cyanobacteria are extinct but APB are abundant. We have

$$\begin{aligned}
0 &= y_1^{(1)}y_2^{(1)} - 1 \\
0 &= x_2^{(1)} \\
0 &= f_1 - y_1^{(1)} - x_1^{(1)}y_1^{(1)}y_2^{(1)} - y_1^{(1)}z^{(1)} \\
0 &= f_2 - y_2^{(1)} - x_1^{(1)}y_1^{(1)}y_2^{(1)} - x_2^{(1)}y_2^{(1)} \\
0 &= ax_2^{(1)}y_2^{(1)} - bz^{(1)} - y_1^{(1)}z^{(1)}
\end{aligned} \tag{17}$$

From Equations (17), we have

$$0 = \left(y_1^{(1)}\right)^2 + (f_2 - f_1)y_1^{(1)} - 1$$

Solving this quadratic equation for $y_1^{(1)}$, keeping the physical solution, and substituting this into Equations (17), we obtain

$$\begin{aligned} x_1^{(1)} &= \frac{f_1 + f_2 - \sqrt{(f_1 - f_2)^2 + 4}}{2} \\ x_2^{(1)} &= 0 \\ y_1^{(1)} &= \frac{f_1 - f_2 + \sqrt{(f_1 - f_2)^2 + 4}}{2} \\ y_2^{(1)} &= \frac{f_2 - f_1 + \sqrt{(f_1 - f_2)^2 + 4}}{2} \\ z^{(1)} &= 0 \end{aligned} \tag{18}$$

Equilibrium E_1 is given by Equations (18).

We set $\bar{x}_1 = 0$ with $\bar{x}_2 > 0$ in Equations (16) to calculate the fixed point for which APB are extinct but cyanobacteria are abundant. We have

$$\begin{aligned} 0 &= x_1^{(2)} \\ 0 &= c y_2^{(2)} - 1 \\ 0 &= f_1 - y_1^{(2)} - x_1^{(2)} y_1^{(2)} y_2^{(2)} - y_1^{(2)} z^{(2)} \\ 0 &= f_2 - y_2^{(2)} - x_1^{(2)} y_1^{(2)} y_2^{(2)} - x_2^{(2)} y_2^{(2)} \\ 0 &= a x_2^{(2)} y_2^{(2)} - b z^{(2)} - y_1^{(2)} z^{(2)} \end{aligned} \tag{19}$$

Simplifying Equations (19), we obtain

$$\begin{aligned} x_1^{(2)} &= 0 \\ x_2^{(2)} &= c f_2 - 1 \\ y_1^{(2)} &= \frac{c(f_1 - b) - a(cf_2 - 1) + \sqrt{[c(f_1 - b) - a(cf_2 - 1)]^2 + 4bc^2 f_1}}{2c} \\ y_2^{(2)} &= \frac{1}{c} \\ z^{(2)} &= \frac{a(cf_2 - 1) - c(f_1 + b) + \sqrt{[c(f_1 - b) - a(cf_2 - 1)]^2 + 4bc^2 f_1}}{2bc} \end{aligned} \tag{20}$$

Equilibrium E_2 is given by Equations (20).

The interior equilibrium corresponds to having both $\bar{x}_1 > 0$ and $\bar{x}_2 > 0$ in Equations (16). We have

$$\begin{aligned} 0 &= \hat{y}_1 \hat{y}_2 - 1 \\ 0 &= c\hat{y}_2 - 1 \\ 0 &= f_1 - \hat{y}_1 - \hat{x}_1 \hat{y}_1 \hat{y}_2 - \hat{y}_1 \hat{z} \\ 0 &= f_2 - \hat{y}_2 - \hat{x}_1 \hat{y}_1 \hat{y}_2 - \hat{x}_2 \hat{y}_2 \\ 0 &= a\hat{x}_2 \hat{y}_2 - b\hat{z} - \hat{y}_1 \hat{z} \end{aligned} \tag{21}$$

The equations for the interior equilibrium are written most simply by using the following defini-

tions:

$$p = c[c - (f_1 - f_2)] - 1 \tag{22}$$

$$q = (b + c)(f_1 - c) - a(cf_2 - 1) \tag{23}$$

$$r = b - c(a - 1) \tag{24}$$

Simplifying Equations (21), we obtain

$$\begin{aligned} \hat{x}_1 &= \frac{q}{r} \\ \hat{x}_2 &= (b + c)\left(\frac{p}{r}\right) \\ \hat{y}_1 &= c \\ \hat{y}_2 &= \frac{1}{c} \\ \hat{z} &= \left(\frac{a}{c}\right)\left(\frac{p}{r}\right) \end{aligned} \tag{25}$$

Equilibrium $\hat{E}$ is given by Equations (25), (22), (23), and (24).

1.4.2 Dynamical stability

For determining the dynamical stability of Equilibrium E_1, we use the following:

$$\begin{aligned} x_1(t) &= x_1^{(1)} + \epsilon(\delta x_1)\exp(\lambda_1 t) \\ x_2(t) &= x_2^{(1)} + \epsilon(\delta x_2)\exp(\lambda_1 t) \\ y_1(t) &= y_1^{(1)} + \epsilon(\delta y_1)\exp(\lambda_1 t) \\ y_2(t) &= y_2^{(1)} + \epsilon(\delta y_2)\exp(\lambda_1 t) \\ z(t) &= z^{(1)} + \epsilon(\delta z)\exp(\lambda_1 t) \end{aligned} \tag{26}$$

Here, we consider that ϵ is arbitrarily small. Substituting Equations (26) into the equation for $\dot{x}_2$ in Equations (8) and setting $u_2 = 0$, we have

$$\lambda_1 \alpha_2 \epsilon(\delta x_2)\exp(\lambda_1 t) = \left[c\left(y_2^{(1)} + \epsilon(\delta y_2)\exp(\lambda_1 t)\right) - 1\right]\epsilon(\delta x_2)\exp(\lambda_1 t)$$

Simplifying and keeping only terms of the lowest order in ϵ, this becomes

$$\lambda_1 = \frac{c y_2^{(1)} - 1}{\alpha_2}$$

If $\lambda_1 < 0$, then E_1 is dynamically stable, while if $\lambda_1 > 0$, then E_1 is dynamically unstable. We can write λ_1 in terms of p using Equation (22):

$$\lambda_1 = \frac{p - (c^2 + 1) + \sqrt{[p - (c^2 + 1)]^2 + 4p}}{2\alpha_2}$$

α_2 is necessarily positive. If $p = 0$, then $\lambda_1 = 0$. If $p < 0$, then $\lambda_1 < 0$. If $p > 0$, then $\lambda_1 > 0$. Therefore, p is sufficient to characterize the dynamical stability of E_1:

- If $p < 0$, then E_1 is dynamically stable.
- If $p > 0$, then E_1 is dynamically unstable.

For determining the dynamical stability of Equilibrium E_2, we use the following:

$$\begin{aligned} x_1(t) &= x_1^{(2)} + \epsilon(\delta x_1)\exp(\lambda_2 t) \\ x_2(t) &= x_2^{(2)} + \epsilon(\delta x_2)\exp(\lambda_2 t) \\ y_1(t) &= y_1^{(2)} + \epsilon(\delta y_1)\exp(\lambda_2 t) \\ y_2(t) &= y_2^{(2)} + \epsilon(\delta y_2)\exp(\lambda_2 t) \\ z(t) &= z^{(2)} + \epsilon(\delta z)\exp(\lambda_2 t) \end{aligned} \tag{27}$$

Here, we consider that ϵ is arbitrarily small. Substituting Equations (27) into the equation for $\dot{x}_1$ in Equations (8) and setting $u_1 = 0$, we have

$$\begin{aligned} \lambda_2\alpha_1\epsilon(\delta x_1)\exp(\lambda_2 t) = &\left[\left(y_1^{(2)} + \epsilon(\delta y_1)\exp(\lambda_2 t)\right)\left(y_2^{(2)} + \epsilon(\delta y_2)\exp(\lambda_2 t)\right) - 1\right] \\ &\times \epsilon(\delta x_1)\exp(\lambda_2 t) \end{aligned}$$

Simplifying and keeping only terms of the lowest order in ϵ, this becomes

$$\lambda_2 = \frac{y_1^{(2)}y_2^{(2)} - 1}{\alpha_1}$$

If $\lambda_2 < 0$, then E_2 is dynamically stable, while if $\lambda_2 > 0$, then E_2 is dynamically unstable. We can write λ_2 in terms of q using Equation (23):

$$\lambda_2 = \frac{q - (c^2 + bf_1) + \sqrt{[q - (c^2 + bf_1)]^2 + 4c^2 q}}{2c^2\alpha_1}$$

α_1 is necessarily positive. If $q = 0$, then $\lambda_2 = 0$. If $q < 0$, then $\lambda_2 < 0$. If $q > 0$, then $\lambda_2 > 0$. Therefore, q is sufficient to characterize the dynamical stability of E_2:

- If $q < 0$, then E_2 is dynamically stable.
- If $q > 0$, then E_2 is dynamically unstable.

Equilibrium $\hat{E}$ can be either stable or unstable. Since there can only be one interior equilibrium, there are two cases for which it exists:

- $p > 0$ and $q > 0$. In this case, $\hat{E}$ is dynamically stable.
- $p < 0$ and $q < 0$. In this case, $\hat{E}$ is dynamically unstable.

1.5 Characterization of the system in steady state

The system in steady state can be understood by considering the values of p and q from Equations (22) and (23), respectively. We have the following possibilities:

- If $p < 0$ and $q > 0$, then APB dominate.
- If $p > 0$ and $q < 0$, then cyanobacteria dominate.
- If $p > 0$ and $q > 0$, then there is stable coexistence.
- If $p < 0$ and $q < 0$, then there is bistability.

For the interior equilibrium to be physical, we must have $\hat{x}_1 > 0$ and $\hat{x}_2 > 0$. From the solutions for $\hat{x}_1$ and $\hat{x}_2$, the key consideration for whether $\hat{E}$ is stable or unstable is then whether $r > 0$ or $r < 0$. If $r > 0$, then it is not possible to have $p < 0$ and $q < 0$, but it is possible to have $p > 0$ and $q > 0$. If $r < 0$, then it is not possible to have $p > 0$ and $q > 0$, but it is possible to have $p < 0$ and $q < 0$. Therefore, for $r > 0$, $\hat{E}$ is necessarily stable, while for $r < 0$, $\hat{E}$ is necessarily unstable.

The possibilities can be written as follows:

- For $r > 0$:
  - If $p < 0$ and $q > 0$, then APB dominate.
  - If $p > 0$ and $q > 0$, then there is stable coexistence.
  - If $p > 0$ and $q < 0$, then cyanobacteria dominate.
- For $r < 0$:
  - If $p < 0$ and $q > 0$, then APB dominate.
  - If $p < 0$ and $q < 0$, then there is bistability.
  - If $p > 0$ and $q < 0$, then cyanobacteria dominate.

1.6 Timing and nature of the GOE

Concerning the GOE, we can ask three basic questions: (1) What was the state of Earth's ecosystem before the GOE? (2) What is the condition that determines when the GOE was triggered? (3) Once the GOE began, was the resulting transition gradual or sudden?

1. Before the GOE, APB dominated over cyanobacteria, so p was necessarily negative.
2. p is a monotonically decreasing function of f_1 and a monotonically increasing function of f_2. Therefore, a decreasing value of f_1 and/or an increasing value of f_2 can cause the pre-GOE equilibrium to become unstable. When f_1 becomes sufficiently small and/or f_2 becomes sufficiently large that $p = 0$, a GOE is triggered. Also, since $p < 0$ when $c = 0$, and since p is a convex, quadratic function of c, an increasing value of c is another possible mechanism for triggering a GOE. p does not depend on a or b, so in the absence of migration of cyanobacteria, an increasing input rate of oxygen or a declining burying rate of oxygen cannot by themselves trigger a GOE.

3. When $p = 0$, the value of r determines the nature of the transition. If $r > 0$, then the GOE was characterized by stable coexistence of APB and cyanobacteria. As conditions continued becoming more favorable to cyanobacteria, the abundance of cyanobacteria gradually increased, while the abundance of APB gradually decreased. If $r < 0$, then the GOE was a rapid and dramatic change in Earth's ecosystem and environment.

A distinguishing feature of a gradual GOE is that, when p becomes positive, the abundance of cyanobacteria increases by many orders of magnitude before there is a significant drop in the abundance of APB. Cyanobacteria and APB both persist at high abundances until q becomes negative, at which point the abundance of APB decreases by many orders of magnitude (**Fig. 3a** and **Fig. S4a**). Moreover, the level of oxygenation depends on the current state of geophysical and biological parameters, not on their histories. In **Fig. S3a**, once f_1 drops below f_1^*, there is a large increase in oxygen abundance, but a subsequent increase in f_1 above f_1^* returns the system to the unoxygenated state.

If, instead, the GOE is a sudden transition, then the abundance of cyanobacteria increases at the same time that the abundance of APB decreases (**Fig. 5a** and **Fig. S5a**). Furthermore, the transition is irreversible—oxygenation is robust to moderate changes in parameter values. There is thus hysteresis. In **Fig. S3b**, once f_1 drops below f_1^*, a GOE results, but a subsequent increase in f_1 above f_1^* does not cause a reversion to the unoxygenated state.

If $a < 1$, then it is impossible to have $r < 0$, regardless of the values of b or c. This is because the small amount of dioxygen that is produced is consumed by rusting without significantly reducing the abundance of iron(II). Therefore, for $a < 1$, the GOE is necessarily gradual.

1.7 Causes of the GOE

The GOE can be caused by any combination of a decrease in f_1, an increase in f_2, or an increase in c. To understand each possibility individually, we consider the effects of holding two of these parameters constant while varying the remaining parameter.

1.7.1 Decreasing f_1

Iron(II) is needed for APB to reproduce. A decrease in the rate of supply of iron(II), f_1, diminishes the rate of reproduction of APB. Setting $p = 0$ in Equation (22) and solving for $f_1 = f_1^*$, we have

$$f_1^* = f_2 + \frac{(c+1)(c-1)}{c} \tag{28}$$

Setting $q = 0$ in Equation (23) and solving for $f_1 = f_1'$, we have

$$f_1' = c + \frac{a(cf_2 - 1)}{b + c} \tag{29}$$

Equations (28) and (29) specify two critical values of f_1. If $f_1 > f_1^*$, then E_1 is stable, while if $f_1 < f_1^*$, then E_1 is unstable. If $f_1 > f_1'$, then E_2 is unstable, while if $f_1 < f_1'$, then E_2 is stable. The effects of a decreasing value of f_1 are shown in **Fig. 3b** and **Fig. 5b**.

1.7.2 Increasing f_2

Phosphate is needed for both APB and cyanobacteria to reproduce. An increase in f_2 leads to an increase in the rates of reproduction of both types of bacteria, but eventually, iron becomes the limiting resource for reproduction of APB. Setting $p = 0$ in Equation (22) and solving for $f_2 = f_2^*$, we have

$$f_2^* = f_1 - \frac{(c+1)(c-1)}{c} \tag{30}$$

Setting $q = 0$ in Equation (23) and solving for $f_2 = f_2'$, we have

$$f_2' = \frac{a + (b+c)(f_1 - c)}{ac} \tag{31}$$

Equations (30) and (31) specify two critical values of f_2. If $f_2 < f_2^*$, then E_1 is stable, while if $f_2 > f_2^*$, then E_1 is unstable. If $f_2 < f_2'$, then E_2 is unstable, while if $f_2 > f_2'$, then E_2 is stable. The effects of an increasing value of f_2 are shown in **Fig. S4b** and **Fig. S5b**.

1.7.3 Increasing c

Another possibility is that the GOE is caused by an increase in c (**Fig. S7a** and **Fig. S8a**). This could be due to geophysical or geochemical changes that are unrelated to the abundances of iron(II) or phosphate. Setting $p = 0$ in Equation (22) and solving for $c = c^*$, we have

$$c^* = \frac{f_1 - f_2 + \sqrt{(f_1 - f_2)^2 + 4}}{2} \tag{32}$$

Setting $q = 0$ in Equation (23) and solving for $c = c'$, we have

$$c' = \frac{f_1 - af_2 - b + \sqrt{(f_1 - af_2 - b)^2 + 4(a + bf_1)}}{2} \tag{33}$$

Equations (32) and (33) specify two critical values of c. If $c < c^*$, then E_1 is stable, while if $c > c^*$, then E_1 is unstable. If $c < c'$, then E_2 is unstable, while if $c > c'$, then E_2 is stable. The effects of an increasing value of c are shown in **Fig. S7b** and **Fig. S8b**.

1.8 Critical values of a and b for Equilibrium E_2

The dynamical stability of E_2 is affected by the values of a and b. Setting $q = 0$ in Equation (23) and solving for $a = a'$, we have

$$a' = (b+c)\left(\frac{f_1 - c}{cf_2 - 1}\right) \tag{34}$$

Equation (34) specifies a critical value of a. If $a < a'$, then E_2 is unstable, while if $a > a'$, then E_2 is stable.

Setting $q = 0$ in Equation (23) and solving for $b = b'$, we have

$$b' = a\left(\frac{cf_2 - 1}{f_1 - c}\right) - c \tag{35}$$

Equation (35) specifies a critical value of b. If $b > b'$, then E_2 is unstable, while if $b < b'$, then E_2 is stable.

1.9 All possible transitions

The following transitions between stable equilibria are possible:

- From E_1 ($p < 0$)
  - $r > 0$ with p increasing: $E_1 \to \hat{E}$ when $p = 0$
  - $r < 0$ with p increasing: $E_1 \to E_2$ when $p = 0$
- From $\hat{E}$ ($p > 0$ and $q > 0$)
  - p decreasing: $\hat{E} \to E_1$ when $p = 0$
  - q decreasing: $\hat{E} \to E_2$ when $q = 0$
- From E_2 ($q < 0$)
  - $r > 0$ with q increasing: $E_2 \to \hat{E}$ when $q = 0$
  - $r < 0$ with q increasing: $E_2 \to E_1$ when $q = 0$

1.10 Migration of cyanobacteria

A decrease in f_1, an increase in f_2, or an increase in c can all cause p—given by Equation (22)—to become positive and trigger a GOE. For an arbitrarily small rate of migration of cyanobacteria, u_2, these causes of the GOE are independent of u_2, a, and b. This is because the accumulation of oxygen due to migration of cyanobacteria is too small to cause any significant amount of rusting of iron.

However, if u_2 is sufficiently large, a is sufficiently large, and b is sufficiently small, then rusting can no longer be neglected in the condition for the GOE. For $\bar{\bar{x}}_1 > 0$, assuming u_1 is arbitrarily small, we set $u_1 = 0$ in Equations (15):

$$\begin{aligned}
0 &= \tilde{y}_1\tilde{y}_2 - 1 \\
0 &= c\tilde{x}_2\tilde{y}_2 - \tilde{x}_2 + u_2 \\
0 &= f_1 - \tilde{y}_1 - \tilde{x}_1\tilde{y}_1\tilde{y}_2 - \tilde{y}_1\tilde{z} \\
0 &= f_2 - \tilde{y}_2 - \tilde{x}_1\tilde{y}_1\tilde{y}_2 - \tilde{x}_2\tilde{y}_2 \\
0 &= a\tilde{x}_2\tilde{y}_2 - b\tilde{z} - \tilde{y}_1\tilde{z}
\end{aligned} \tag{36}$$

Eliminating $\tilde{x}_1$, $\tilde{x}_2$, $\tilde{y}_1$, and $\tilde{z}$ from Equations (36), we have

$$0 = \psi(f_1, f_2, c, a, b, u_2, \tilde{y}_2) \tag{37}$$

$\psi(f_1, f_2, c, a, b, u_2, \tilde{y}_2)$ in Equation (37) is given by

$$\psi(f_1, f_2, c, a, b, u_2, \tilde{y}_2) = k_0 + k_1\tilde{y}_2 + k_2\left(\tilde{y}_2\right)^2 + k_3\left(\tilde{y}_2\right)^3 + k_4\left(\tilde{y}_2\right)^4$$

The coefficients are given by

$$
\begin{aligned}
k_0 &= 1 \\
k_1 &= f_2 - f_1 + b - c \\
k_2 &= (f_2 - f_1)(b - c) - bc - 1 + (a-1)u_2 \\
k_3 &= c - b - bc(f_2 - f_1) - bu_2 \\
k_4 &= bc
\end{aligned}
$$

For understanding the possibility of a GOE, we must consider the values of $\tilde{y}_2$ obtained from solving $\psi(f_1, f_2, c, a, b, u_2, \tilde{y}_2) = 0$. If the GOE is gradual, then there is one stable equilibrium. As f_1 decreases, f_2 increases, c increases, a increases, b decreases, or u_2 increases, this fixed point corresponds to a decreasing equilibrium value of $\tilde{x}_1$ and an increasing equilibrium value of $\tilde{x}_2$ (**Fig. 4**).

If the GOE is sudden, then as f_1 decreases, f_2 increases, c increases, a increases, b decreases, or u_2 increases, an unstable interior equilibrium appears (**Fig. 6**). Just before a sudden rise in atmospheric oxygen occurs, there are two fixed points of the dynamics that are arbitrarily close to each other in phase space. If parameters change slightly, then there can be a saddle-node bifurcation, where $\psi(f_1, f_2, c, a, b, u_2, \tilde{y}_2)$ has a double root for $\tilde{y}_2$. When this double root exists, the discriminant of the quartic polynomial $\psi(f_1, f_2, c, a, b, u_2, \tilde{y}_2)$, denoted by $\Delta(f_1, f_2, c, a, b, u_2)$, is equal to zero.

A decreasing f_1, an increasing f_2, or an increasing c can cause a GOE, leading to the following three equations:

$$0 = \Delta(f_1^*, f_2, c, a, b, u_2) \tag{38}$$
$$0 = \Delta(f_1, f_2^*, c, a, b, u_2) \tag{39}$$
$$0 = \Delta(f_1, f_2, c^*, a, b, u_2) \tag{40}$$

The critical values of f_1, f_2, and c for triggering a GOE are solutions to Equations (38), (39), and (40), respectively. f_1^*, f_2^*, and c^* are approximately given by Equations (28), (30), and (32), respectively, if u_2 is sufficiently small, a is sufficiently small, and b is sufficiently large (**Fig. S6**). Moreover, since $\Delta(f_1, f_2, c, a, b, u_2)$ is also a function of a, b, and u_2, a sufficiently large value of a, a sufficiently small value of b, and a sufficiently large value of u_2 are each capable of triggering a GOE. This leads to the following three equations:

$$0 = \Delta(f_1, f_2, c, a^*, b, u_2) \tag{41}$$
$$0 = \Delta(f_1, f_2, c, a, b^*, u_2) \tag{42}$$
$$0 = \Delta(f_1, f_2, c, a, b, u_2^*) \tag{43}$$

The critical values of a, b, and u_2 for triggering a GOE are solutions to Equations (41), (42), and (43), respectively.

For a declining b to be able to cause a GOE, a must be sufficiently large. This leads to the following equation:

$$0 = \Delta(f_1, f_2, c, \eta, 0, u_2) \tag{44}$$

The critical value of a, denoted by η, for which a declining b is able to cause a GOE is a solution to Equation (44). If $a > \eta$, then the critical value of b for triggering a GOE is given by b^*. But if $a < \eta$, then a declining b cannot trigger a GOE.

We can solve approximately for η. In Equations (36), we set $b = 0$, and we make the substitution $\tilde{x}_1 \to \chi$, where $\chi = x_1^{(1)}$ from Equations (18):

$$\chi = \frac{f_1 + f_2 - \sqrt{(f_1 - f_2)^2 + 4}}{2} \tag{45}$$

We have

$$\begin{aligned} 0 &\approx \tilde{y}_1' \tilde{y}_2' - 1 \\ 0 &\approx c\tilde{x}_2' \tilde{y}_2' - \tilde{x}_2' + u_2 \\ 0 &\approx f_1 - \tilde{y}_1' - \chi \tilde{y}_1' \tilde{y}_2' - \tilde{y}_1' \tilde{z}' \\ 0 &\approx a\tilde{x}_2' \tilde{y}_2' - \tilde{y}_1' \tilde{z}' \end{aligned} \tag{46}$$

Eliminating $\tilde{x}_2'$, $\tilde{y}_1'$, and $\tilde{z}'$ from Equations (46), we have

$$0 \approx 1 - (f_1 - \chi + c)\tilde{y}_2' + [c(f_1 - \chi) + au_2] (\tilde{y}_2')^2 \tag{47}$$

The discriminant of the quadratic polynomial on the right-hand side of Equation (47) is equal to zero if $a = \eta$, where

$$\eta = \frac{(f_1 - \chi - c)^2}{4u_2} \tag{48}$$

We can also solve approximately for a^* and b^*. To do this, we set $a = \eta$ and $\tilde{y}_1 = \Upsilon$ in Equations (46), we eliminate $\tilde{x}_2$, $\tilde{y}_2$, and $\tilde{z}$, we substitute Equation (48), and we solve for Υ:

$$\Upsilon = \frac{f_1 - \chi + c}{2} \tag{49}$$

Then, in Equations (36), we make the substitution $\tilde{x}_1 \to \chi$, and in the rusting terms $(\tilde{y}_1 \tilde{z})$ in the third and fifth of Equations (36), we make the substitution $\tilde{y}_1 \to \Upsilon$:

$$\begin{aligned} 0 &\approx \tilde{y}_1 \tilde{y}_2 - 1 \\ 0 &\approx c\tilde{x}_2 \tilde{y}_2 - \tilde{x}_2 + u_2 \\ 0 &\approx f_1 - \tilde{y}_1 - \chi \tilde{y}_1 \tilde{y}_2 - \Upsilon \tilde{z} \\ 0 &\approx a\tilde{x}_2 \tilde{y}_2 - (b + \Upsilon)\tilde{z} \end{aligned} \tag{50}$$

Eliminating $\tilde{x}_2$, $\tilde{y}_1$, and $\tilde{z}$ from Equations (50), we have

$$0 \approx (b + \Upsilon) - (f_1 - \chi + c)(b + \Upsilon)\tilde{y}_2 + [c(f_1 - \chi)(b + \Upsilon) + \Upsilon a u_2] (\tilde{y}_2)^2 \tag{51}$$

The discriminant of the quadratic polynomial on the right-hand side of Equation (51) is equal to zero if $a = \alpha$, where

$$\alpha = \eta \left(1 + \frac{b}{\Upsilon}\right) \tag{52}$$

Similarly, this discriminant is equal to zero if $b = \beta$, where

$$\beta = (a - \eta) \left(\frac{\Upsilon}{\eta}\right) \tag{53}$$

The critical values a^* and b^* for triggering a GOE are approximately given by α and β, respectively, from Equations (52) and (53), with Υ given by Equation (49), η given by Equation (48), and χ given by Equation (45) (**Fig. S11**).

If a GOE is caused by an increase in a, a reduction in b, or an increase in u_2, then the resulting transformation is necessarily discontinuous. This is because E_2—characterized by abundance of oxygen—gains stability first, and as a continues to rise, b continues to drop, or u_2 continues to rise, the unstable interior equilibrium moves toward E_1 in phase space until a saddle-node bifurcation occurs.

Fig. S9 shows a GOE caused by a rising production rate of oxygen. **Fig. S10** shows a GOE caused by a declining burial rate of oxygen.

1.11 Corrections to the fixed points due to migration

For $u_1 > 0$ and $u_2 > 0$, the fixed points, $(\bar{\bar{x}}_1, \bar{\bar{x}}_2, \bar{\bar{y}}_1, \bar{\bar{y}}_2, \bar{\bar{z}})$, are given by Equations (15). For sufficiently small values of u_1 and u_2, we can treat u_1 and u_2 as perturbations. We can express $(\bar{\bar{x}}_1, \bar{\bar{x}}_2, \bar{\bar{y}}_1, \bar{\bar{y}}_2, \bar{\bar{z}})$ as

$$\begin{aligned} \bar{\bar{x}}_1 &= \bar{x}_1 + \delta x_1 \\ \bar{\bar{x}}_2 &= \bar{x}_2 + \delta x_2 \\ \bar{\bar{y}}_1 &= \bar{y}_1 + \delta y_1 \\ \bar{\bar{y}}_2 &= \bar{y}_2 + \delta y_2 \\ \bar{\bar{z}} &= \bar{z} + \delta z \end{aligned} \tag{54}$$

Here, $(\delta x_1, \delta x_2, \delta y_1, \delta y_2, \delta z)$ represent corrections to $(\bar{x}_1, \bar{x}_2, \bar{y}_1, \bar{y}_2, \bar{z})$ due to the migration rates, u_1 and u_2. Substituting Equations (54) into Equations (15), we obtain

$$\begin{pmatrix} -u_1 \\ -u_2 \\ 0 \\ 0 \\ 0 \end{pmatrix} \approx \\ \begin{pmatrix} (\bar{y}_1\bar{y}_2 - 1) & 0 & \bar{x}_1\bar{y}_2 & \bar{x}_1\bar{y}_1 & 0 \\ 0 & (c\bar{y}_2 - 1) & 0 & c\bar{x}_2 & 0 \\ -\bar{y}_1\bar{y}_2 & 0 & -(1 + \bar{z} + \bar{x}_1\bar{y}_2) & -\bar{x}_1\bar{y}_1 & -\bar{y}_1 \\ -\bar{y}_1\bar{y}_2 & -\bar{y}_2 & -\bar{x}_1\bar{y}_2 & -(1 + \bar{x}_2 + \bar{x}_1\bar{y}_1) & 0 \\ 0 & a\bar{y}_2 & -\bar{z} & a\bar{x}_2 & -(b + \bar{y}_1) \end{pmatrix} \begin{pmatrix} \delta x_1 \\ \delta x_2 \\ \delta y_1 \\ \delta y_2 \\ \delta z \end{pmatrix} \tag{55}$$

Equations (55) can be solved to obtain approximations for $(\delta x_1, \delta x_2, \delta y_1, \delta y_2, \delta z)$.

1.12 Effects of migration rates

For sufficiently small values of u_1 and u_2, the calculations for timing and duration of the GOE are robust to changes in the migration rates. **Fig. S12** shows a continuous transition for different values of u_1 and u_2. **Fig. S13** shows a discontinuous transition for different values of u_1 and u_2.

The value of u_1 determines the magnitude of the decline in APB, x_1. This is given by

$$\frac{x_1^{(1)}}{\delta x_1\big|_{E_2}} = \frac{x_1^{(1)}\left(1 - y_1^{(2)} y_2^{(2)}\right)}{u_1} \tag{56}$$

From Equation (56), the magnitude of the decline in APB is inversely proportional to u_1.

The value of u_2 determines the magnitude of the rise in cyanobacteria, x_2, and the magnitude of the rise in oxygen, z. These are given by

$$\frac{x_2^{(2)}}{\delta x_2\big|_{E_1}} = \frac{x_2^{(2)}\left(1 - c y_2^{(1)}\right)}{u_2} \tag{57}$$

$$\frac{z^{(2)}}{\delta z\big|_{E_1}} = \frac{z^{(2)}\left(1 - c y_2^{(1)}\right)\left(b + y_1^{(1)}\right)}{a y_2^{(1)} u_2} \tag{58}$$

From Equations (57) and (58), the magnitude of the rise in cyanobacteria and the magnitude of the rise in oxygen are inversely proportional to u_2.

2 Supplementary Note 2: Bounded bacterial growth rates

In Equations (1), the growth rates of APB and cyanobacteria are unbounded. The growth rates of both types of bacteria are proportional to the abundance of phosphate, and the growth rate of APB is also proportional to the abundance of iron(II). It is more realistic to model these growth rates as being bounded. To do this, we consider the following equations:

$$\begin{aligned}
\frac{dX_1}{dT} &= C_1 X_1 \left(\frac{Y_1}{1 + M Y_1}\right)\left(\frac{Y_2}{1 + N_1 Y_2}\right) - D_1 X_1 + U_1 \\
\frac{dX_2}{dT} &= C_2 X_2 \left(\frac{Y_2}{1 + N_2 Y_2}\right) - D_2 X_2 + U_2 \\
\frac{dY_1}{dT} &= F_1 - G_1 Y_1 - H X_1 \left(\frac{Y_1}{1 + M Y_1}\right)\left(\frac{Y_2}{1 + N_1 Y_2}\right) - R_1 Y_1 Z \\
\frac{dY_2}{dT} &= F_2 - G_2 Y_2 - H_1 X_1 \left(\frac{Y_1}{1 + M Y_1}\right)\left(\frac{Y_2}{1 + N_1 Y_2}\right) - H_2 X_2 \left(\frac{Y_2}{1 + N_2 Y_2}\right) \\
\frac{dZ}{dT} &= A X_2 \left(\frac{Y_2}{1 + N_2 Y_2}\right) - B Z - R Y_1 Z
\end{aligned} \tag{59}$$

For small Y_1, the factor $Y_1/(1 + MY_1)$ is approximately equal to Y_1, but as Y_1 becomes large, this factor approaches a maximum value of $1/M$. For small Y_2, the factor $Y_2/(1 + N_1 Y_2)$ is approximately equal to Y_2, but as Y_2 becomes large, this factor approaches a maximum value of $1/N_1$. For small Y_2, the factor $Y_2/(1 + N_2 Y_2)$ is approximately equal to Y_2, but as Y_2 becomes large, this factor approaches a maximum value of $1/N_2$. To simplify Equations (59), we make the

following definitions:

$$\begin{aligned} m &\equiv \frac{D_1 M}{k C_1} \\ n_1 &\equiv k N_1 \\ n_2 &\equiv k N_2 \end{aligned} \tag{60}$$

Substituting Equations (6), (7), and (60) into Equations (59), we obtain

$$\begin{aligned} \alpha_1 \dot{x}_1 &= x_1 \left(\frac{y_1}{1+my_1}\right)\left(\frac{y_2}{1+n_1 y_2}\right) - x_1 + u_1 \\ \alpha_2 \dot{x}_2 &= c x_2 \left(\frac{y_2}{1+n_2 y_2}\right) - x_2 + u_2 \\ \beta_1 \dot{y}_1 &= f_1 - y_1 - x_1 \left(\frac{y_1}{1+my_1}\right)\left(\frac{y_2}{1+n_1 y_2}\right) - y_1 z \\ \beta_2 \dot{y}_2 &= f_2 - y_2 - x_1 \left(\frac{y_1}{1+my_1}\right)\left(\frac{y_2}{1+n_1 y_2}\right) - x_2 \left(\frac{y_2}{1+n_2 y_2}\right) \\ \dot{z} &= a x_2 \left(\frac{y_2}{1+n_2 y_2}\right) - bz - y_1 z \end{aligned} \tag{61}$$

For studying the ecological dynamics, without loss of generality, we consider Equations (61).

The fixed points of the ecological dynamics are obtained from the steady state of Equations (61):

$$\begin{aligned} 0 &= \bar{\bar{x}}_1 \left(\frac{\bar{\bar{y}}_1}{1+m\bar{\bar{y}}_1}\right)\left(\frac{\bar{\bar{y}}_2}{1+n_1 \bar{\bar{y}}_2}\right) - \bar{\bar{x}}_1 + u_1 \\ 0 &= c\bar{\bar{x}}_2 \left(\frac{\bar{\bar{y}}_2}{1+n_2 \bar{\bar{y}}_2}\right) - \bar{\bar{x}}_2 + u_2 \\ 0 &= f_1 - \bar{\bar{y}}_1 - \bar{\bar{x}}_1 \left(\frac{\bar{\bar{y}}_1}{1+m\bar{\bar{y}}_1}\right)\left(\frac{\bar{\bar{y}}_2}{1+n_1 \bar{\bar{y}}_2}\right) - \bar{\bar{y}}_1 \bar{\bar{z}} \\ 0 &= f_2 - \bar{\bar{y}}_2 - \bar{\bar{x}}_1 \left(\frac{\bar{\bar{y}}_1}{1+m\bar{\bar{y}}_1}\right)\left(\frac{\bar{\bar{y}}_2}{1+n_1 \bar{\bar{y}}_2}\right) - \bar{\bar{x}}_2 \left(\frac{\bar{\bar{y}}_2}{1+n_2 \bar{\bar{y}}_2}\right) \\ 0 &= a\bar{\bar{x}}_2 \left(\frac{\bar{\bar{y}}_2}{1+n_2 \bar{\bar{y}}_2}\right) - b\bar{\bar{z}} - \bar{\bar{y}}_1 \bar{\bar{z}} \end{aligned} \tag{62}$$

We solve for the fixed points of Equations (61) without loss of generality by considering Equations (62).

Setting $u_1 = u_2 = 0$ in Equations (62), we have

$$\begin{aligned}
0 &= \left[\left(\frac{\bar{y}_1}{1+m\bar{y}_1}\right)\left(\frac{\bar{y}_2}{1+n_1\bar{y}_2}\right) - 1\right]\bar{x}_1 \\
0 &= \left[c\left(\frac{\bar{y}_2}{1+n_2\bar{y}_2}\right) - 1\right]\bar{x}_2 \\
0 &= f_1 - \bar{y}_1 - \bar{x}_1\left(\frac{\bar{y}_1}{1+m\bar{y}_1}\right)\left(\frac{\bar{y}_2}{1+n_1\bar{y}_2}\right) - \bar{y}_1\bar{z} \\
0 &= f_2 - \bar{y}_2 - \bar{x}_1\left(\frac{\bar{y}_1}{1+m\bar{y}_1}\right)\left(\frac{\bar{y}_2}{1+n_1\bar{y}_2}\right) - \bar{x}_2\left(\frac{\bar{y}_2}{1+n_2\bar{y}_2}\right) \\
0 &= a\bar{x}_2\left(\frac{\bar{y}_2}{1+n_2\bar{y}_2}\right) - b\bar{z} - \bar{y}_1\bar{z}
\end{aligned} \tag{63}$$

We set $\bar{x}_2 = 0$ with $\bar{x}_1 > 0$ in Equations (63) to calculate the fixed point for which cyanobacteria are extinct but APB are abundant. We have

$$\begin{aligned}
0 &= \left(\frac{y_1^{(1)}}{1+my_1^{(1)}}\right)\left(\frac{y_2^{(1)}}{1+n_1y_2^{(1)}}\right) - 1 \\
0 &= x_2^{(1)} \\
0 &= f_1 - y_1^{(1)} - x_1^{(1)}\left(\frac{y_1^{(1)}}{1+my_1^{(1)}}\right)\left(\frac{y_2^{(1)}}{1+n_1y_2^{(1)}}\right) - y_1^{(1)}z^{(1)} \\
0 &= f_2 - y_2^{(1)} - x_1^{(1)}\left(\frac{y_1^{(1)}}{1+my_1^{(1)}}\right)\left(\frac{y_2^{(1)}}{1+n_1y_2^{(1)}}\right) - x_2^{(1)}\left(\frac{y_2^{(1)}}{1+n_2y_2^{(1)}}\right) \\
0 &= ax_2^{(1)}\left(\frac{y_2^{(1)}}{1+n_2y_2^{(1)}}\right) - bz^{(1)} - y_1^{(1)}z^{(1)}
\end{aligned} \tag{64}$$

From Equations (64), we have

$$0 = (1-mn_1)\left(y_1^{(1)}\right)^2 + [(f_2-f_1)(1-mn_1) - m - n_1]y_1^{(1)} - [1 + (f_2-f_1)n_1]$$

Solving this quadratic equation for $y_1^{(1)}$, keeping the physical solution, and substituting this into Equations (64), we obtain Equilibrium E_1:

$$\begin{aligned}
x_1^{(1)} &= \frac{(f_1+f_2)(1-mn_1) - m - n_1 - \sqrt{[(f_1-f_2)(1-mn_1)+m-n_1]^2+4}}{2(1-mn_1)} \\
x_2^{(1)} &= 0 \\
y_1^{(1)} &= \frac{(f_1-f_2)(1-mn_1) + m + n_1 + \sqrt{[(f_1-f_2)(1-mn_1)+m-n_1]^2+4}}{2(1-mn_1)} \\
y_2^{(1)} &= \frac{(f_2-f_1)(1-mn_1) + m + n_1 + \sqrt{[(f_1-f_2)(1-mn_1)+m-n_1]^2+4}}{2(1-mn_1)} \\
z^{(1)} &= 0
\end{aligned} \tag{65}$$

We set $\bar{x}_1 = 0$ with $\bar{x}_2 > 0$ in Equations (63) to calculate the fixed point for which APB are extinct but cyanobacteria are abundant. We have

$$
\begin{aligned}
0 &= x_1^{(2)} \\
0 &= c\left(\frac{y_2^{(2)}}{1+n_2 y_2^{(2)}}\right) - 1 \\
0 &= f_1 - y_1^{(2)} - x_1^{(2)}\left(\frac{y_1^{(2)}}{1+m y_1^{(2)}}\right)\left(\frac{y_2^{(2)}}{1+n_1 y_2^{(2)}}\right) - y_1^{(2)} z^{(2)} \\
0 &= f_2 - y_2^{(2)} - x_1^{(2)}\left(\frac{y_1^{(2)}}{1+m y_1^{(2)}}\right)\left(\frac{y_2^{(2)}}{1+n_1 y_2^{(2)}}\right) - x_2^{(2)}\left(\frac{y_2^{(2)}}{1+n_2 y_2^{(2)}}\right) \\
0 &= a x_2^{(2)}\left(\frac{y_2^{(2)}}{1+n_2 y_2^{(2)}}\right) - b z^{(2)} - y_1^{(2)} z^{(2)}
\end{aligned}
\tag{66}
$$

Simplifying Equations (66), we obtain Equilibrium E_2:

$$
\begin{aligned}
x_1^{(2)} &= 0 \\
x_2^{(2)} &= \frac{c[f_2(c-n_2)-1]}{c-n_2} \\
y_1^{(2)} &= \frac{1}{2(c-n_2)}\Big[(c-n_2)(f_1-b) - a[(c-n_2)f_2 - 1] \\
&\quad + \sqrt{\{(c-n_2)(f_1-b) - a[(c-n_2)f_2-1]\}^2 + 4b(c-n_2)^2 f_1}\Big] \\
y_2^{(2)} &= \frac{1}{c-n_2} \\
z^{(2)} &= \frac{1}{2b(c-n_2)}\Big[a[(c-n_2)f_2-1] - (c-n_2)(f_1+b) \\
&\quad + \sqrt{\{(c-n_2)(f_1-b) - a[(c-n_2)f_2-1]\}^2 + 4b(c-n_2)^2 f_1}\Big]
\end{aligned}
\tag{67}
$$

The interior equilibrium corresponds to having both $\bar{x}_1 > 0$ and $\bar{x}_2 > 0$ in Equations (63). We have

$$
\begin{aligned}
0 &= \left(\frac{\hat{y}_1}{1+m\hat{y}_1}\right)\left(\frac{\hat{y}_2}{1+n_1\hat{y}_2}\right) - 1 \\
0 &= c\left(\frac{\hat{y}_2}{1+n_2\hat{y}_2}\right) - 1 \\
0 &= f_1 - \hat{y}_1 - \hat{x}_1\left(\frac{\hat{y}_1}{1+m\hat{y}_1}\right)\left(\frac{\hat{y}_2}{1+n_1\hat{y}_2}\right) - \hat{y}_1\hat{z} \\
0 &= f_2 - \hat{y}_2 - \hat{x}_1\left(\frac{\hat{y}_1}{1+m\hat{y}_1}\right)\left(\frac{\hat{y}_2}{1+n_1\hat{y}_2}\right) - \hat{x}_2\left(\frac{\hat{y}_2}{1+n_2\hat{y}_2}\right) \\
0 &= a\hat{x}_2\left(\frac{\hat{y}_2}{1+n_2\hat{y}_2}\right) - b\hat{z} - \hat{y}_1\hat{z}
\end{aligned}
\tag{68}
$$

The equations for the interior equilibrium are written most simply by defining p', q', and r'. For p', we have

$$p' = (c - n_2)\{(c - n_2 + n_1) - (f_1 - f_2)[1 - m(c - n_2 + n_1)]\} - [1 - m(c - n_2 + n_1)] \quad (69)$$

For q', we have

$$\begin{aligned} q' = \{&b[1 - m(c - n_2 + n_1)] + (c - n_2 + n_1)\}\{f_1[1 - m(c - n_2 + n_1)] - (c - n_2 + n_1)\} \\ &\times (c - n_2) \\ &- a[(c - n_2)f_2 - 1][1 - m(c - n_2 + n_1)](c - n_2 + n_1) \end{aligned} \quad (70)$$

For r', we have

$$r' = b[1 - m(c - n_2 + n_1)] - (c - n_2 + n_1)(a - 1) \quad (71)$$

Simplifying Equations (68), we obtain Equilibrium $\hat{E}$:

$$\begin{aligned} \hat{x}_1 &= \left(\frac{1}{(c - n_2)[1 - m(c - n_2 + n_1)]}\right)\left(\frac{q'}{r'}\right) \\ \hat{x}_2 &= \left(\frac{c\{b[1 - m(c - n_2 + n_1)] + (c - n_2 + n_1)\}}{(c - n_2)[1 - m(c - n_2 + n_1)]}\right)\left(\frac{p'}{r'}\right) \\ \hat{y}_1 &= \frac{c - n_2 + n_1}{1 - m(c - n_2 + n_1)} \\ \hat{y}_2 &= \frac{1}{c - n_2} \\ \hat{z} &= \left(\frac{a}{c - n_2}\right)\left(\frac{p'}{r'}\right) \end{aligned} \quad (72)$$

2.1 Dynamical stability

For determining the dynamical stability of Equilibrium E_1, we use the following:

$$\begin{aligned} x_1(t) &= x_1^{(1)} + \epsilon(\delta x_1)\exp(\lambda_1 t) \\ x_2(t) &= x_2^{(1)} + \epsilon(\delta x_2)\exp(\lambda_1 t) \\ y_1(t) &= y_1^{(1)} + \epsilon(\delta y_1)\exp(\lambda_1 t) \\ y_2(t) &= y_2^{(1)} + \epsilon(\delta y_2)\exp(\lambda_1 t) \\ z(t) &= z^{(1)} + \epsilon(\delta z)\exp(\lambda_1 t) \end{aligned} \quad (73)$$

Here, we consider that ϵ is arbitrarily small. Substituting Equations (73) into the equation for $\dot{x}_2$ in Equations (61) and setting $u_2 = 0$, we have

$$\lambda_1 \alpha_2 \epsilon(\delta x_2)\exp(\lambda_1 t) = \left[\frac{c\left(y_2^{(1)} + \epsilon(\delta y_2)\exp(\lambda_1 t)\right)}{1 + n_2\left(y_2^{(1)} + \epsilon(\delta y_2)\exp(\lambda_1 t)\right)} - 1\right]\epsilon(\delta x_2)\exp(\lambda_1 t)$$

Simplifying and keeping only terms of the lowest order in ϵ, this becomes

$$\lambda_1 = \frac{1}{\alpha_2}\left(\frac{c y_2^{(1)}}{1 + n_2 y_2^{(1)}} - 1\right)$$

This can be rewritten as

$$\lambda_1 = \frac{(c - n_2) y_2^{(1)} - 1}{\left(1 + n_2 y_2^{(1)}\right)\alpha_2}$$

If $\lambda_1 < 0$, then E_1 is dynamically stable, while if $\lambda_1 > 0$, then E_1 is dynamically unstable. We can write λ_1 in terms of p' using Equation (69):

$$\begin{aligned}
\lambda_1 = &\left(\frac{1}{2(1 - mn_1)[1 - m(c - n_2 + n_1)]\left(1 + n_2 y_2^{(1)}\right)\alpha_2}\right) \\
&\times \Bigg\{(1 - mn_1)p' - \{(c - n_2)^2 + [1 - m(c - n_2 + n_1)]^2\} \\
&+ \text{sgn}(c - n_2)\text{sgn}(1 - m(c - n_2 + n_1)) \\
&\times \Bigg[\Big((1 - mn_1)p' - \{(c - n_2)^2 + [1 - m(c - n_2 + n_1)]^2\}\Big)^2 \\
&+ 4(1 - mn_1)[1 - m(c - n_2 + n_1)]^2 p'\Bigg]^{1/2}\Bigg\}
\end{aligned} \tag{74}$$

The first of Equations (64) yields

$$1 - mn_1 > 0$$

The dynamical stability of E_1 is determined from Equation (74) as follows:

- If $c - n_2 < 0$, then E_1 is dynamically stable.
- If $c - n_2 > 0$ and $1 - m(c - n_2 + n_1) > 0$ and $p' < 0$, then E_1 is dynamically stable.
- If $c - n_2 > 0$ and $1 - m(c - n_2 + n_1) > 0$ and $p' > 0$, then E_1 is dynamically unstable.
- If $1 - m(c - n_2 + n_1) < 0$, then E_1 is dynamically unstable.

For determining the dynamical stability of Equilibrium E_2, we use the following:

$$\begin{aligned}
x_1(t) &= x_1^{(2)} + \epsilon(\delta x_1)\exp(\lambda_2 t) \\
x_2(t) &= x_2^{(2)} + \epsilon(\delta x_2)\exp(\lambda_2 t) \\
y_1(t) &= y_1^{(2)} + \epsilon(\delta y_1)\exp(\lambda_2 t) \\
y_2(t) &= y_2^{(2)} + \epsilon(\delta y_2)\exp(\lambda_2 t) \\
z(t) &= z^{(2)} + \epsilon(\delta z)\exp(\lambda_2 t)
\end{aligned} \tag{75}$$

Here, we consider that ϵ is arbitrarily small. Substituting Equations (75) into the equation for $\dot{x}_1$ in Equations (61) and setting $u_1 = 0$, we have

$$\lambda_2 \alpha_1 \epsilon(\delta x_1) \exp(\lambda_2 t) = \left[\left(\frac{y_1^{(2)} + \epsilon(\delta y_1)\exp(\lambda_2 t)}{1 + m\left(y_1^{(2)} + \epsilon(\delta y_1)\exp(\lambda_2 t)\right)} \right) \times \left(\frac{y_2^{(2)} + \epsilon(\delta y_2)\exp(\lambda_2 t)}{1 + n_1\left(y_2^{(2)} + \epsilon(\delta y_2)\exp(\lambda_2 t)\right)} \right) - 1 \right] \epsilon(\delta x_1)\exp(\lambda_2 t)$$

Simplifying and keeping only terms of the lowest order in ϵ, this becomes

$$\lambda_2 = \frac{1}{\alpha_1}\left[\left(\frac{y_1^{(2)}}{1 + m y_1^{(2)}}\right)\left(\frac{y_2^{(2)}}{1 + n_1 y_2^{(2)}}\right) - 1\right]$$

This can be rewritten as

$$\lambda_2 = \frac{(1 - m n_1) y_1^{(2)} y_2^{(2)} - m y_1^{(2)} - n_1 y_2^{(2)} - 1}{\left(1 + m y_1^{(2)}\right)\left(1 + n_1 y_2^{(2)}\right)\alpha_1}$$

If $\lambda_2 < 0$, then E_2 is dynamically stable, while if $\lambda_2 > 0$, then E_2 is dynamically unstable. We can write λ_2 in terms of q' using Equation (70):

$$\begin{aligned}\lambda_2 = &\left(\frac{1}{2(c-n_2)^2(c-n_2+n_1)\left(1+my_1^{(2)}\right)\left(1+n_1y_2^{(2)}\right)\alpha_1}\right) \\ &\times \Bigg\{ q' - (c-n_2)\{(c-n_2+n_1)^2 + bf_1[1-m(c-n_2+n_1)]^2\} \\ &+ \operatorname{sgn}(1-m(c-n_2+n_1)) \\ &\times \left[\left(q' - (c-n_2)\{(c-n_2+n_1)^2 + bf_1[1-m(c-n_2+n_1)]^2\}\right)^2 \right. \\ &\left. + 4(c-n_2)(c-n_2+n_1)^2 q'\right]^{1/2}\Bigg\}\end{aligned} \tag{76}$$

The second of Equations (66) yields

$$c - n_2 > 0$$

The dynamical stability of E_2 is determined from Equation (76) as follows:

- If $1 - m(c - n_2 + n_1) < 0$, then E_2 is dynamically stable.
- If $1 - m(c - n_2 + n_1) > 0$ and $q' < 0$, then E_2 is dynamically stable.
- If $1 - m(c - n_2 + n_1) > 0$ and $q' > 0$, then E_2 is dynamically unstable.

Equilibrium $\hat{E}$ can be either stable or unstable. Since there can only be one interior equilibrium, there are two cases for which it exists:

- E_1 and E_2 are both dynamically unstable. In this case, $\hat{E}$ is dynamically stable.
- E_1 and E_2 are both dynamically stable. In this case, $\hat{E}$ is dynamically unstable.

2.2 Characterization of the interior equilibrium

For the interior equilibrium to be physical, we must have $\hat{x}_1 > 0$ and $\hat{x}_2 > 0$. The equations for $\hat{y}_1$ and $\hat{y}_2$ from Equations (72) also require that $c - n_2 > 0$ and $1 - m(c - n_2 + n_1) > 0$. From the solutions for $\hat{x}_1$ and $\hat{x}_2$, the key consideration for whether $\hat{E}$ is stable or unstable is then whether $r' > 0$ or $r' < 0$. If $r' > 0$, then it is not possible to have $p' < 0$ and $q' < 0$, but it is possible to have $p' > 0$ and $q' > 0$. If $r' < 0$, then it is not possible to have $p' > 0$ and $q' > 0$, but it is possible to have $p' < 0$ and $q' < 0$. Therefore, for $r' > 0$, $\hat{E}$ is necessarily stable, while for $r' < 0$, $\hat{E}$ is necessarily unstable.

2.3 Simplified model: $n_1 = n_2 = n$

The analysis is simplified by setting $n_1 = n_2 = n$. For the dynamics, Equations (61) become

$$\begin{aligned}
\alpha_1 \dot{x}_1 &= x_1 \left(\frac{y_1}{1+my_1}\right)\left(\frac{y_2}{1+ny_2}\right) - x_1 + u_1 \\
\alpha_2 \dot{x}_2 &= cx_2 \left(\frac{y_2}{1+ny_2}\right) - x_2 + u_2 \\
\beta_1 \dot{y}_1 &= f_1 - y_1 - x_1 \left(\frac{y_1}{1+my_1}\right)\left(\frac{y_2}{1+ny_2}\right) - y_1 z \\
\beta_2 \dot{y}_2 &= f_2 - y_2 - x_1 \left(\frac{y_1}{1+my_1}\right)\left(\frac{y_2}{1+ny_2}\right) - x_2 \left(\frac{y_2}{1+ny_2}\right) \\
\dot{z} &= ax_2 \left(\frac{y_2}{1+ny_2}\right) - bz - y_1 z
\end{aligned} \tag{77}$$

Equations (65) become

$$\begin{aligned}
x_1^{(1)} &= \frac{(f_1+f_2)(1-mn) - m - n - \sqrt{[(f_1-f_2)(1-mn)+m-n]^2+4}}{2(1-mn)} \\
x_2^{(1)} &= 0 \\
y_1^{(1)} &= \frac{(f_1-f_2)(1-mn) + m + n + \sqrt{[(f_1-f_2)(1-mn)+m-n]^2+4}}{2(1-mn)} \\
y_2^{(1)} &= \frac{(f_2-f_1)(1-mn) + m + n + \sqrt{[(f_1-f_2)(1-mn)+m-n]^2+4}}{2(1-mn)} \\
z^{(1)} &= 0
\end{aligned} \tag{78}$$

Equilibrium E_1 is given by Equations (78).

Equations (67) become

$$
\begin{aligned}
x_1^{(2)} &= 0 \\
x_2^{(2)} &= \frac{c[f_2(c-n)-1]}{c-n} \\
y_1^{(2)} &= \frac{1}{2(c-n)}\Bigg[(c-n)(f_1-b)-a[(c-n)f_2-1] \\
&\quad + \sqrt{\{(c-n)(f_1-b)-a[(c-n)f_2-1]\}^2+4b(c-n)^2f_1}\Bigg] \\
y_2^{(2)} &= \frac{1}{c-n} \\
z^{(2)} &= \frac{1}{2b(c-n)}\Bigg[a[(c-n)f_2-1]-(c-n)(f_1+b) \\
&\quad + \sqrt{\{(c-n)(f_1-b)-a[(c-n)f_2-1]\}^2+4b(c-n)^2f_1}\Bigg]
\end{aligned}
\tag{79}
$$

Equilibrium E_2 is given by Equations (79).

Equation (69) becomes

$$p' = (c-n)[c-(f_1-f_2)(1-mc)]-(1-mc) \tag{80}$$

Equation (70) becomes

$$q' = [b(1-mc)+c][f_1(1-mc)-c](c-n)-a[(c-n)f_2-1](1-mc)c \tag{81}$$

Equation (71) becomes

$$r' = b(1-mc)-c(a-1) \tag{82}$$

Equations (72) become

$$
\begin{aligned}
\hat{x}_1 &= \left(\frac{1}{(c-n)(1-mc)}\right)\left(\frac{q'}{r'}\right) \\
\hat{x}_2 &= \left(\frac{c[b(1-mc)+c]}{(c-n)(1-mc)}\right)\left(\frac{p'}{r'}\right) \\
\hat{y}_1 &= \frac{c}{1-mc} \\
\hat{y}_2 &= \frac{1}{c-n} \\
\hat{z} &= \left(\frac{a}{c-n}\right)\left(\frac{p'}{r'}\right)
\end{aligned}
\tag{83}
$$

Equilibrium $\hat{E}$ is given by Equations (83), (80), (81), and (82).

2.3.1 Effects of m and n on the GOE

Bounds on the bacterial growth rates do not change the roles of f_1 and f_2 in triggering a GOE. Similarly to p, p' only depends on the difference $f_1 - f_2$. Therefore, the difference $f_1 - f_2$ determines when the GOE is initiated.

In **Fig. S14(a)**, $f_1^* - f_2$ is plotted versus c for $n = 0$ and for different values of m. For $c \ll 1/m$, m does not significantly change the condition for initiating a GOE. For larger values of c, the value of $f_1^* - f_2$ is augmented compared with the case $m = 0$. If $c > 1/m$, then the fixed point for which APB dominate is necessarily unstable. In **Fig. S14(b)**, $f_1^* - f_2$ is plotted versus c for $m = 0$ and for different values of n. For $c \gg n$, n does not significantly change the condition for initiating a GOE. For smaller values of c, the value of $f_1^* - f_2$ is reduced compared with the case $n = 0$. If $c < n$, then the fixed point for which APB dominate is necessarily stable.

For nonzero values of m and n, the GOE can be continuous or discontinuous. **Fig. S15** shows a continuous transition, and **Fig. S16** shows a discontinuous transition.

3 Supplementary Note 3: Dynamics of organic carbon

When APB and cyanobacteria die, they leave behind organic matter. The carbon in this organic matter can be buried in sediments and thereby removed from the system. Another possibility is that organic carbon reacts with oxygen in the atmosphere. To understand how aerobic decomposition of organic carbon affects the GOE, we consider the following equations:

$$\begin{aligned}
\frac{dX_1}{dT} &= C_1 X_1 Y_1 Y_2 - D_1 X_1 + U_1 \\
\frac{dX_2}{dT} &= C_2 X_2 Y_2 - D_2 X_2 + U_2 \\
\frac{dY_1}{dT} &= F_1 - G_1 Y_1 - H X_1 Y_1 Y_2 - R_1 Y_1 Z \\
\frac{dY_2}{dT} &= F_2 - G_2 Y_2 - H_1 X_1 Y_1 Y_2 - H_2 X_2 Y_2 \\
\frac{dZ}{dT} &= A X_2 Y_2 - B Z - R Y_1 Z - E W Z \\
\frac{dW}{dT} &= J_1 X_1 + J_2 X_2 - S W - V W Z
\end{aligned} \tag{84}$$

In Equations (84), W is organic carbon. Organic carbon is produced when APB die at rate $J_1 X_1$ and when cyanobacteria die at rate $J_2 X_2$. Organic carbon is removed via reaction with dioxygen at rate VWZ, and dioxygen is correspondingly removed at rate EWZ. Organic carbon is removed by other means at rate SW.

Equations (84) can be rewritten as

$$\begin{aligned}
\left(\frac{1}{D_1}\right)\frac{d}{dT}\left(\frac{kHX_1}{G_1}\right) &= \left(\frac{kHX_1}{G_1}\right)\left(\frac{kC_1Y_1}{D_1}\right)\left(\frac{Y_2}{k}\right) - \frac{kHX_1}{G_1} + \frac{kHU_1}{D_1G_1} \\
\left(\frac{1}{D_2}\right)\frac{d}{dT}\left(\frac{H_2X_2}{G_2}\right) &= \left(\frac{kC_2}{D_2}\right)\left(\frac{H_2X_2}{G_2}\right)\left(\frac{Y_2}{k}\right) - \frac{H_2X_2}{G_2} + \frac{H_2U_2}{D_2G_2} \\
\left(\frac{1}{G_1}\right)\frac{d}{dT}\left(\frac{kC_1Y_1}{D_1}\right) &= \frac{kC_1F_1}{D_1G_1} - \frac{kC_1Y_1}{D_1} \\
&\quad - \left(\frac{kHX_1}{G_1}\right)\left(\frac{kC_1Y_1}{D_1}\right)\left(\frac{Y_2}{k}\right) - \left(\frac{kC_1Y_1}{D_1}\right)\left(\frac{R_1Z}{G_1}\right) \\
\left(\frac{1}{G_2}\right)\frac{d}{dT}\left(\frac{Y_2}{k}\right) &= \frac{F_2}{kG_2} - \frac{Y_2}{k} \\
&\quad - \left(\frac{kHX_1}{G_1}\right)\left(\frac{kC_1Y_1}{D_1}\right)\left(\frac{Y_2}{k}\right) - \left(\frac{H_2X_2}{G_2}\right)\left(\frac{Y_2}{k}\right) \\
\left(\frac{kC_1}{D_1R}\right)\frac{d}{dT}\left(\frac{R_1Z}{G_1}\right) &= \left(\frac{k^2AC_1G_2R_1}{D_1G_1H_2R}\right)\left(\frac{H_2X_2}{G_2}\right)\left(\frac{Y_2}{k}\right) \\
&\quad - \left(\frac{kBC_1}{D_1R}\right)\left(\frac{R_1Z}{G_1}\right) - \left(\frac{kC_1Y_1}{D_1}\right)\left(\frac{R_1Z}{G_1}\right) \\
&\quad - \left(\frac{kC_1EW}{D_1R}\right)\left(\frac{R_1Z}{G_1}\right) \\
\left(\frac{R_1}{G_1V}\right)\frac{d}{dT}\left(\frac{kC_1EW}{D_1R}\right) &= \left(\frac{C_1EJ_1R_1}{D_1HRV}\right)\left(\frac{kHX_1}{G_1}\right) + \left(\frac{kC_1EG_2J_2R_1}{D_1G_1H_2RV}\right)\left(\frac{H_2X_2}{G_2}\right) \\
&\quad - \left(\frac{R_1S}{G_1V}\right)\left(\frac{kC_1EW}{D_1R}\right) \\
&\quad - \left(\frac{kC_1EW}{D_1R}\right)\left(\frac{R_1Z}{G_1}\right)
\end{aligned} \tag{85}$$

To simplify Equations (85), we make the following definitions:

$$\begin{aligned}
j_1 &\equiv \frac{C_1EJ_1R_1}{D_1HRV} \\
j_2 &\equiv \frac{kC_1EG_2J_2R_1}{D_1G_1H_2RV} \\
s &\equiv \frac{R_1S}{G_1V} \\
w &\equiv \frac{kC_1EW}{D_1R}
\end{aligned} \tag{86}$$

We also define

$$\Omega \equiv \frac{R_1}{\gamma G_1V} \tag{87}$$

Substituting Equations (6), (7), (86), and (87) into Equations (85), we obtain

$$\begin{aligned}
\alpha_1\dot{x}_1 &= x_1y_1y_2 - x_1 + u_1 \\
\alpha_2\dot{x}_2 &= cx_2y_2 - x_2 + u_2 \\
\beta_1\dot{y}_1 &= f_1 - y_1 - x_1y_1y_2 - y_1z \\
\beta_2\dot{y}_2 &= f_2 - y_2 - x_1y_1y_2 - x_2y_2 \\
\dot{z} &= ax_2y_2 - bz - y_1z - wz \\
\Omega\dot{w} &= j_1x_1 + j_2x_2 - sw - wz
\end{aligned} \tag{88}$$

Equations (88) describe, without loss of generality, the ecological dynamics with organic carbon.

The fixed points of the ecological dynamics are obtained from the steady state of Equations (88):

$$\begin{aligned}
0 &= \bar{\bar{x}}_1\bar{\bar{y}}_1\bar{\bar{y}}_2 - \bar{\bar{x}}_1 + u_1 \\
0 &= c\bar{\bar{x}}_2\bar{\bar{y}}_2 - \bar{\bar{x}}_2 + u_2 \\
0 &= f_1 - \bar{\bar{y}}_1 - \bar{\bar{x}}_1\bar{\bar{y}}_1\bar{\bar{y}}_2 - \bar{\bar{y}}_1\bar{\bar{z}} \\
0 &= f_2 - \bar{\bar{y}}_2 - \bar{\bar{x}}_1\bar{\bar{y}}_1\bar{\bar{y}}_2 - \bar{\bar{x}}_2\bar{\bar{y}}_2 \\
0 &= a\bar{\bar{x}}_2\bar{\bar{y}}_2 - b\bar{\bar{z}} - \bar{\bar{y}}_1\bar{\bar{z}} - \bar{\bar{w}}\bar{\bar{z}} \\
0 &= j_1\bar{\bar{x}}_1 + j_2\bar{\bar{x}}_2 - s\bar{\bar{w}} - \bar{\bar{w}}\bar{\bar{z}}
\end{aligned} \tag{89}$$

Setting $u_1 = u_2 = 0$ in Equations (89), we have

$$\begin{aligned}
0 &= (\bar{y}_1\bar{y}_2 - 1)\bar{x}_1 \\
0 &= (c\bar{y}_2 - 1)\bar{x}_2 \\
0 &= f_1 - \bar{y}_1 - \bar{x}_1\bar{y}_1\bar{y}_2 - \bar{y}_1\bar{z} \\
0 &= f_2 - \bar{y}_2 - \bar{x}_1\bar{y}_1\bar{y}_2 - \bar{x}_2\bar{y}_2 \\
0 &= a\bar{x}_2\bar{y}_2 - b\bar{z} - \bar{y}_1\bar{z} - \bar{w}\bar{z} \\
0 &= j_1\bar{x}_1 + j_2\bar{x}_2 - s\bar{w} - \bar{w}\bar{z}
\end{aligned} \tag{90}$$

For Equilibrium E_1, $x_2^{(1)} = z^{(1)} = 0$. Thus, from Equation (22), p is sufficient to characterize the dynamical stability of E_1:

- If $p < 0$, then E_1 is dynamically stable.
- If $p > 0$, then E_1 is dynamically unstable.

To determine the dynamical stability of Equilibrium E_2, we consider Equilibrium $\hat{E}$ in the limit that $\hat{x}_1$ is arbitrarily small. From Equations (90), Equilibrium $\hat{E}$ is given by

$$\begin{aligned}
0 &= \hat{y}_1\hat{y}_2 - 1 \\
0 &= c\hat{y}_2 - 1 \\
0 &= f_1 - \hat{y}_1 - \hat{x}_1\hat{y}_1\hat{y}_2 - \hat{y}_1\hat{z} \\
0 &= f_2 - \hat{y}_2 - \hat{x}_1\hat{y}_1\hat{y}_2 - \hat{x}_2\hat{y}_2 \\
0 &= a\hat{x}_2\hat{y}_2 - b\hat{z} - \hat{y}_1\hat{z} - \hat{w}\hat{z} \\
0 &= j_1\hat{x}_1 + j_2\hat{x}_2 - s\hat{w} - \hat{w}\hat{z}
\end{aligned} \tag{91}$$

Setting $\hat{x}_1 = 0$ in Equations (91), we have

$$
\begin{aligned}
0 &= \hat{y}_1^{(2)}\hat{y}_2^{(2)} - 1 \\
0 &= c\hat{y}_2^{(2)} - 1 \\
0 &= f_1 - \hat{y}_1^{(2)} - \hat{y}_1^{(2)}\hat{z}^{(2)} \\
0 &= f_2 - \hat{y}_2^{(2)} - \hat{x}_2^{(2)}\hat{y}_2^{(2)} \\
0 &= a\hat{x}_2^{(2)}\hat{y}_2^{(2)} - b\hat{z}^{(2)} - \hat{y}_1^{(2)}\hat{z}^{(2)} - \hat{w}^{(2)}\hat{z}^{(2)} \\
0 &= j_2\hat{x}_2^{(2)} - s\hat{w}^{(2)} - \hat{w}^{(2)}\hat{z}^{(2)}
\end{aligned}
\tag{92}
$$

Using q from Equation (23), we define

$$\tilde{q} = q[f_1 - c(1 - s)] + j_2c(cf_2 - 1)(f_1 - c) \tag{93}$$

Eliminating $\hat{x}_2^{(2)}$, $\hat{y}_1^{(2)}$, $\hat{y}_2^{(2)}$, $\hat{z}^{(2)}$, and $\hat{w}^{(2)}$ from Equations (92), we obtain $\tilde{q} = 0$. This indicates that, if $f_1 > c$, then $\tilde{q}$ from Equation (93) is sufficient to characterize the dynamical stability of E_2. We have the following conditions:

- If $f_1 < c$, or if $f_1 > c$ and $\tilde{q} < 0$, then E_2 is dynamically stable.
- If $f_1 > c$ and $\tilde{q} > 0$, then E_2 is dynamically unstable.

We can further determine how organic carbon affects the duration of the GOE. Using r from Equation (24), we define

$$\tilde{r} = r + \frac{j_2c^2(cf_2 - 1)}{sc^2 + cf_2 - 1} \tag{94}$$

Considering that $f_1 > c$, the solution to $p = 0$ and $\tilde{q} = 0$ is that $\tilde{r} = 0$. This indicates that $\tilde{r}$ from Equation (94) determines whether the GOE is gradual or sudden:

- If $\tilde{r} > 0$, then the transition is gradual.
- If $\tilde{r} < 0$, then the transition is sudden.

3.1 Effects of s on the GOE

The parameter s is the rate of burial of organic carbon. If organic carbon is immediately buried in sediments, then s is arbitrarily large, and organic carbon has no effect on the GOE. For finite values of s, some organic carbon reacts with oxygen during aerobic decomposition, thereby removing oxygen from the atmosphere. Organic carbon thus represents one of many potential sinks for oxygen. Since p is independent of sinks for oxygen, the rate of burial of organic carbon is not a central consideration for determining when the GOE begins. It can, however, affect the duration of the GOE.

Fig. S17(a) shows a continuous transition for a decreasing value of f_1 and for a finite value of s. In **Fig. S17(b)**, the value of s is increased, and the transition is instead discontinuous. In both cases, oxygen levels rise substantially around time $t = t^*$.
